# Japanese encephalitis virus hijacks the host purine biosynthetic network to promote viral replication in neurons

**DOI:** 10.1371/journal.ppat.1014335

**Published:** 2026-07-07

**Authors:** Jun Gu, Chencheng Fan, Shengxian Xiang, Ling’en Yang, Jia Liu, Youhui Si, Bibo Zhu, Yingjie Sun, Chan Ding, Shengbo Cao, Jing Ye

**Affiliations:** 1 National Key Laboratory of Agricultural Microbiology, Huazhong Agricultural University, Wuhan, Hubei, China; 2 Hubei Hongshan Laboratory, Wuhan, Hubei, China; 3 Frontiers Science Center for Animal Breeding and Sustainable Production, College of Veterinary Medicine, Huazhong Agricultural University, Wuhan, Hubei, China; 4 Key Laboratory of Preventive Veterinary Medicine in Hubei Province, Wuhan, Hubei, China; 5 Key Laboratory of Animal Pathogen Infection and Immunology of Fujian Province, College of Animal Sciences, Fujian Agriculture and Forestry University, Fuzhou, China; 6 Department of Avian Infectious Diseases, Shanghai Veterinary Research Institute, Chinese Academy of Agricultural Science, Shanghai, China; 7 School of Agriculture and Biology, Shanghai Jiao Tong University, Shanghai, China; Oregon Health & Science University, UNITED STATES OF AMERICA

## Abstract

Japanese encephalitis virus (JEV) is an important neurotropic orthoflavivirus that poses a threat to both human and animal health. However, the mechanism underlying its rapid replication in the central nervous system (CNS) remains poorly understood. In this study, we conducted metabolomic profiling of JEV-infected mouse brains and neurons, revealing a profound reprogramming of central carbon metabolism, particularly an enhancement in nucleotide synthesis. Integrated multi-omics analyses confirmed that JEV infection transcriptionally upregulates key enzymes involved in *de novo* purine biosynthesis (DNPB), one-carbon (1C) metabolism, and the pentose phosphate pathway (PPP) in neurons. Pharmacological inhibition of the core DNPB enzymes potently suppressed JEV replication in neurons and reduced both viral loads and neuroinflammation in JEV-infected mice, suggesting the essential role of DNPB in JEV replication within CNS. Mechanistically, we delineated the critical functions of both the non-oxidative PPP and MTHFD2-mediated 1C metabolism, which jointly supply essential precursors, such as ribose-5-phosphate and formyl groups, for the *de novo* biosynthesis of purines required for viral RNA replication. These findings unveil a strategy by which JEV co-opts the host's purine biosynthetic machinery to fulfill the nucleotide demands for its genomic replication, establishing DNPB and its supporting pathways as promising therapeutic targets for infections caused by JEV and other neurotropic viruses.

## Author summary

Japanese encephalitis virus (JEV) must hijack neuronal resources to replicate, particularly within the resource-limited environment of the brain. However, the precise metabolic mechanisms it employs within neurons remain poorly understood, hindering the development of targeted therapy. We found that JEV utilizes a novel strategy by compelling infected neurons to activate *de novo* purine biosynthesis, a pathway that neurons rarely engage, to supply nucleotides for its genome synthesis. This process is fueled by the coordinated viral hijacking of two supporting pathways: one-carbon metabolism and the non-oxidative pentose phosphate pathway, which provide essential formyl groups and ribose-5-phosphate, respectively. Our findings redefine the understanding of neurotropic virus-host interactions by revealing a precise program of neuronal anabolic reprogramming. Furthermore, we identified key enzymes within this network, GART and IMPDH, as promising therapeutic targets. This work establishes a host-directed metabolic inhibition strategy, offering a new potential approach against Japanese encephalitis and similar central nervous system infections.

## Introduction

Japanese encephalitis virus (JEV), a member of the *Orthoflavivirus* genus within family *Flaviviridae*, is a significant zoonotic neurotropic virus with a single-stranded positive-sense RNA genome [1]. Upon infection, JEV can penetrate the blood-brain barrier (BBB) and replicate efficiently within neurons, causing Japanese encephalitis, a severe condition associated with a mortality rate of approximately 30% and long-term neurological complications in 30–50% of survivors [2]. Infections in farm animals, particularly pigs, also lead to reproductive disorders, resulting in substantial economic losses [3,4]. JEV is prevalent in South Asia, the Western Pacific, and parts of northern Australia [5], with an estimated 68,000 cases reported annually [6,7]. Recent reports have indicated its emergence in previously unaffected areas, such as Ningxia and Xinjiang in China [8,9], raising concerns about its expanding geographical range, which may be influenced by global warming, agricultural development, and increased migration of humans and pigs [10]. Vaccination is available and recommended for individuals at high risk of exposure, but it offers no therapeutic benefit once infection is established. Current management of JEV infection remains limited to supportive care due to the lack of targeted antiviral agents, underscoring the urgent need for research into viral replication mechanisms to identify molecular targets and develop effective therapeutics.

As obligate intracellular parasitic organisms, viruses rely on host cells for energy, molecular precursors, and other essential components required for replication [11]. Over time, various viruses have developed sophisticated mechanisms to interface with and hijack the metabolic machinery of host cells, including glycolysis, fatty acid synthesis, and glutaminolysis [12], thereby completing their replication cycles. Consequently, metabolic interventions could inhibit viral replication by targeting the host cell’s metabolic processes. Direct inhibition of nucleotide biosynthesis enzymes is a well-established strategy for combating viral infections. A series of purine and pyrimidine synthesis inhibitors have demonstrated antiviral efficacy against multiple viruses [13,14].

The brain, despite constituting only 2% of body weight, exhibits the highest energy demand in the human body, consuming approximately 20% of the body's glucose and oxygen to sustain the high activity levels of its neurons [15,16]. Glucose serves as the primary energy substrate for the central nervous system (CNS), fueling approximately 95% of ATP production in the brain [17]. Following its transport across the BBB, glucose is predominantly metabolized via glycolysis and oxidative phosphorylation (OXPHOS) to generate ATP [18,19], thereby fueling both neurons and glial cells. Beyond this primary energy-yielding metabolism, glucose is further metabolized through the pentose phosphate pathway (PPP) to generate reducing power in the form of NADPH, which is essential for combating oxidative stress and facilitating lipid synthesis [20]. Additionally, the BBB allows the transport of nucleobases and nucleosides into the brain via specific transporters, thereby supporting nucleotide salvage synthesis [21]. This salvage pathway efficiently utilizes the breakdown products of nucleotides to meet the high energy demands of the brain, allowing for rapid regeneration of nucleotides, particularly ATP. Critically, salvage synthesis can swiftly ramp up activity during cellular stress when substrate demand rises, by capitalizing on accelerated nucleotide turnover. This resynthesis route is both energetically favorable and metabolically efficient [22]. In contrast, the *de novo* synthesis pathway builds nucleotides through a multi-step and energy-consuming enzymatic process that initiates with small-molecule precursors. The *de novo* purine biosynthesis (DNPB) pathway comprises ten sequential enzymatic reactions that convert phosphoribosyl pyrophosphate (PRPP) into inosine monophosphate (IMP). This process requires precursors derived from multiple metabolic branches, including glutamine, glycine, aspartate, and 10-formyl-tetrahydrofolate (10-formyl-THF). The pathway is tightly regulated at the committing step catalyzed by PPAT, and the enzymes involved are known to assemble into a dynamic multi-enzyme complex termed the ‘purinosome’ to enhance metabolic flux during periods of high purine demand [23]. In the developing mammalian brain, purine metabolism exhibits a characteristic spatiotemporal shift: the *de novo* pathway is highly active during early embryonic stages to support neural stem/progenitor cell proliferation, whereas the salvage pathway becomes predominant around the neonatal period [24]. Consequently, it is well-established that the mature mammalian CNS exhibits minimal *de novo* nucleotide synthesis, predominantly relying on salvage pathways to satisfy its nucleotide requirements [22,25,26].

To gain comprehensive insights into JEV-induced metabolic reprogramming in CNS, we conducted metabolic analyses on JEV-infected mouse brain and the neuronal cell line N2a utilizing LC-MS technology. Our study showed that JEV hijacks central carbon metabolism (CCM) in neurons to fuel viral replication, with a critical dependency on DNPB. Pharmacological inhibition of DNPB demonstrated potential therapeutic efficacy, as evidenced by prolonged survival, reduced viral loads in brain tissue, and attenuated neuroinflammation in JEV-infected mice. Furthermore, JEV exploits the non-oxidative PPP and MTHFD2-mediated one-carbon metabolism to synergistically provide ribose-5-phosphate and formyl groups for nucleotide biosynthesis. Collectively, these findings reveal an unexpected role of DNPB in JEV replication within CNS and indicate that targeting DNPB could be a potential therapeutic strategy against Japanese encephalitis.

## Results

### JEV infection induces broad metabolic reprogramming in mouse brain

**Fig 1:**
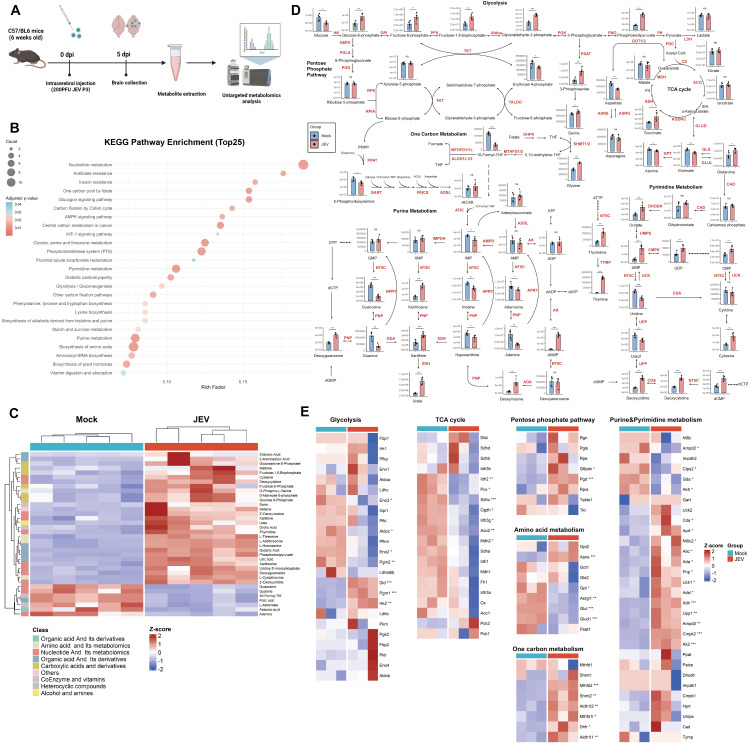
JEV infection induces significant metabolic reprogramming in mice brains. **(A)** Schematic diagram of the experimental design. Six-week-old female C57BL/6 mice were intracranially injected with 200 PFU of JEV P3 strain or DMEM (n = 5). Brains were harvested at 5 dpi for LC-MS-based metabolomic analysis. Created in BioRender. Gu, J. (2026) https://BioRender.com/f2uz4b3. **(B)** KEGG pathway enrichment analysis of significantly altered metabolites shows the top 25 enriched metabolic pathways. **(C)** Heatmap of Z-scores showing the relative abundance of key metabolites from the top 25 enriched pathways in mock and JEV-infected brains (n = 5). **(D)** Integrated metabolic network analysis. Schematic representation of key metabolic pathways, including glycolysis, TCA cycle, pentose phosphate pathway, one-carbon metabolism, nucleotide metabolism, and amino acid metabolism, with metabolite abundance changes overlaid. Data are presented as mean ± s.e.m.; statistical significance was determined using two-tailed unpaired Student’s t-tests (n = 5 mice per group, *p < 0.05, **p < 0.01, ***p < 0.001). **(E)** Transcriptomic analysis of enzyme-encoding genes in key metabolic pathways. The heatmap displays transcriptional changes of genes involved in indicated pathways in mouse brain between JEV-infected and mock-infected groups. Statistical significance was determined using two-tailed unpaired Student’s t-tests (n = 3 mice per group, *p < 0.05, **p < 0.01, ***p < 0.001).

To gain biological insights into the reprogramming of key host metabolic pathways induced by JEV-infection, six-week-old female C57BL/6 mice were intracranially injected with 200 PFU of JEV P3 strain or an equal volume DMEM as a control. At 5 dpi, the mouse brains were harvested and then subjected to metabolic analysis using LC-MS (Fig 1A). Principal component analysis (PCA) showed distinct clustering between the mock-infected controls and the JEV-infected mouse brains, indicating substantial metabolic differences between the two groups (S1A Fig). Metabolomic profiling of brain tissues revealed profound metabolic remodeling upon JEV infection, with 260 metabolites exhibiting significant alterations (VIP > 1, p value<0.05, |Log_2_FC| > 1) (S1B and S1C Fig, S3 Table). Among these, 204 metabolites were found to be upregulated, while 56 were downregulated in the brains of JEV-infected mice compared to the mock-infected controls (S1B and S1C Fig). The Kyoto Encyclopedia of Genes and Genomes (KEGG) pathway enrichment analysis identified the top 25 significantly enriched metabolic pathways, including nucleotide metabolism (encompassing purine and pyrimidine metabolism), one-carbon (1C) metabolism, glycolysis, and amino acid metabolism (Fig 1B). Heatmap visualization further revealed global metabolic shifts associated with these enriched pathways, characterized by marked accumulation of glycolytic intermediates and significant alterations in nucleotide precursors (Fig 1C).

To visualize the metabolic crosstalk and identify critical nodes, we constructed an integrated network map synthesizing glycolysis, TCA cycle, PPP, one-carbon metabolism, nucleotide metabolism, and key amino acid branches, overlaying metabolite abundance (Fig 1D, S3 Table). In parallel, to explore potential regulatory mechanisms underlying the observed metabolic alterations, we integrated these metabolomic findings with our previous transcriptomic data [27], focusing specifically on enzyme-encoding genes within enriched pathways (Fig 1E). This integrated analysis revealed a profound reprogramming of CCM characterized by the accumulation of glycolytic intermediates, such as glucose-6-phosphate, fructose-6-phosphate, fructose-1,6-bisphosphate, glyceraldehyde-3-phosphate, and phosphoenolpyruvate, despite significant glucose depletion and stable lactate levels (Fig 1D). This metabolic rewiring coincided with the transcriptional upregulation of the rate-limiting enzyme *Hk2*, indicating *Hk2*-driven substrate channeling that enhances the glycolytic pathway (Fig 1E). Analysis of TCA cycle metabolites revealed a significant accumulation of succinate, while citrate, isocitrate, and malate exhibited no significant changes (Fig 1D). Concurrently, transcriptomic profiling showed a significant downregulation of core TCA enzymes: *Ogdh*, *Sdhc*, *Pcx*, *Idh2*, *Idh3g*, *Aco2* and *Mdh2* (Fig 1E). Among PPP metabolites analyzed, erythrose-4-phosphate (E4P) was uniquely downregulated, while other intermediates showed no significant alterations (Fig 1D). Concurrently, transcriptional upregulation of *G6pdx* and *Pgd* was observed (Fig 1E). This coordinated enhancement of oxidative PPP enzymes, along with E4P depletion suggests amplified flux through the oxidative branch and attenuated activity in the non-oxidative branch in infected mouse brains. Depleted aspartate and glutamate abundances, alongside elevated glutamine levels (Fig 1D), aligned with transcriptional alterations of *Asns*, *Asrgl1*, *Glud*, and *Glul* (Fig 1E)*.* Furthermore, the observed upregulation in the abundance of 3-phosphoserine and serine indicates enhanced *de novo* serine synthesis in the JEV-infected mouse brain (Fig 1D). We also found increased transcriptional levels of key 1C metabolism enzymes, including *Dhfr*, *Shmt2*, *Mthfd2*, *Mthfd1l*, and *Aldh1l1/2* (Fig 1E). Notably, the upregulation of *Shmt2*, which catalyzes the direct conversion of serine to glycine while generating 5,10-methylene-THF, aligns with the observed increase in glycine abundance (Fig 1D and 1E). This further corroborates enhanced 1C metabolism. However, the levels of 10-Formyl-THF were significantly decreased (Fig 1D). This apparent contradiction may be attributed to the heightened consumption of 10-formyl-THF, potentially driven by hyperactive purine biosynthesis which utilizes this cofactor as a critical formyl donor.

In our metabolomic analysis, intermediates of nucleotide metabolism exhibited deeply pronounced alterations (Fig 1D). Though transcriptional analysis showed no significant changes in the expression of critical *de novo* pyrimidine biosynthesis enzyme genes *Dhodh* and *Cad*, key intermediates of *de novo* pyrimidine biosynthesis, including orotate and UMP were significantly upregulated compared to uninfected controls (Fig 1E). This seems to be distinct from the brain’s generally limited capacity for *de novo* nucleotide synthesis under normal physiological conditions. Concurrently, we observed a significant decrease in the levels of uracil and uridine, whereas cytosine, thymine, thymidine, deoxyuridine, and deoxycytidine were significantly increased (Fig 1D). This reduction of uracil and uridine levels correlated with upregulated transcription of key salvage pathway enzyme genes *Uck2* and *Upp1* (Fig 1E). These findings suggest that JEV infection enhances both *de novo* pyrimidine biosynthesis and salvage pathways, ensuring sufficient nucleotide supply.

Furthermore, the transcriptomic analysis revealed that JEV infection significantly enhances purine catabolism, as evidenced by the elevated expression of key enzyme genes governing nucleotide breakdown and interconversion, including *Pnp*, *Ada*, *Ampd3*, *Nt5c2*, and crucially, *Xdh* (Fig 1E). This indicates an accelerated degradation of nucleotides and nucleosides toward terminal metabolites. Metabolomic profiling corroborated this shift, showing a marked accumulation of xanthine/urate (Fig 1D). Despite this catabolic bias, the upregulation of salvage enzyme APRT and the interconversion hub NT5C2 suggests a compensatory mechanism aimed at recycling residual purine bases and nucleosides. This salvage effort likely contributes to stabilizing cellular AMP/GMP pools amid heightened purine demand during infection. While *de novo* purine biosynthesis enzymes ADSL and ATIC were transcriptionally induced (Fig 1E), the early precursor 5-phosphoribosylamine decreased (p < 0.01) without detectable expansion of the IMP pool (Fig 1D), implying substrate consumption exceeding synthetic capacity. Finally, induction of mitochondrial *Ak2* likely preserved energy homeostasis by dynamically balancing ATP/ADP/AMP ratios (Fig 1E), thereby supporting the high-flux purine metabolic state during JEV infection. Collectively, these data demonstrate that JEV systematically hijacks host metabolic networks in the brain, orchestrating a multi-layered reprogramming of central carbon and nucleotide metabolism.

### Targeted metabolomics reveals rewiring of central carbon metabolism in JEV-infected neurons

**Fig 2:**
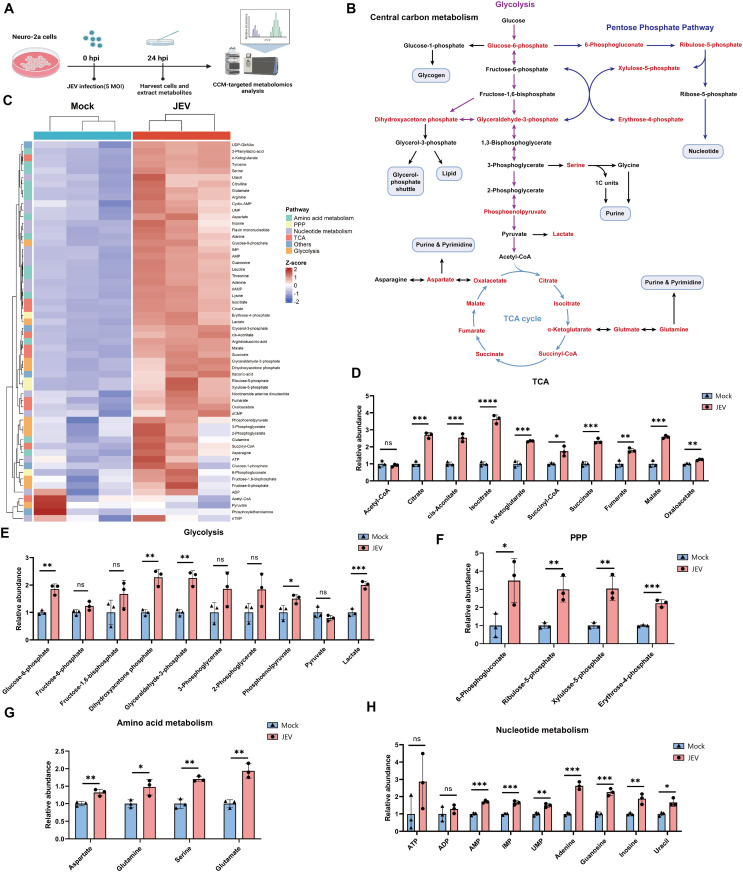
Targeted metabolomics identifies JEV-induced rewiring of central carbon metabolism in N2a Cells. **(A)** Schematic of the experimental workflow for targeted metabolomic analysis. N2a cells were infected or mock-infected with JEV P3 strain at an MOI of 5. The cells were collected at 24 hpi for CCM-targeted metabolomic analysis. Created in BioRender. Gu, J. (2026) https://BioRender.com/f2uz4b3. **(B)** Schematic representation of integrated central carbon metabolism pathways. Metabolites that were significantly upregulated in JEV-infected N2a cells compared to mock-infected controls are highlighted in red. No metabolites were significantly downregulated in this targeted dataset. **(C)** Heatmap visualization of detected cellular metabolites in JEV-infected vs. mock-infected N2a cells at 24 hpi (n = 3). **(D-H)** Comparative analysis of metabolic alterations in key pathways, including TCA cycle **(D)**, glycolysis **(E)**, pentose phosphate pathway (PPP) **(F)**, amino acid metabolism **(G)**, and nucleotide metabolism **(H)**. Data are presented as mean ± s.e.m. (n = 3). Statistical significance was determined using two-tailed unpaired Student's t-tests (*p < 0.05, **p < 0.01, ***p < 0.001, ****p < 0.0001).

To validate the widespread perturbations across multiple metabolic networks identified by the untargeted metabolomic analysis, we performed a targeted metabolomic analysis in mock and JEV-infected N2a, a mouse neuronal cell line (Fig 2A). This analysis specifically profiled multiple core intermediates of CCM, which integrates glycolysis, TCA cycle, and PPP (Fig 2B), serving dual roles in energy production and in supplying precursors for biosynthesis [28]. The reproducibility of the metabolomics dataset was confirmed by PCA, which showed tight clustering of biological replicates and clear separation between mock‑ and JEV‑infected groups (S1D Fig). Targeted metabolomic profiling revealed extensive remodeling of CCM induced by JEV infection, involving a substantial number of differentially regulated core intermediates (Fig 2C, S4 Table). The TCA cycle exhibited specific dysregulation, characterized by a coordinated accumulation of downstream intermediates (including malate, cis-aconitate, and α-ketoglutarate), while acetyl-CoA levels remained unchanged (Fig 2D). Concurrently, there was an accumulation of several glycolytic intermediates, including G6P, G3P, and 2-phosphoglycerate (2PG) (Fig 2E), indicating an enhancement of glycolysis. Among these intermediates, G3P can be converted into ribose-5-phosphate (R5P) via carbon-shuffling reactions involving sedoheptulose-7-phosphate (S7P) in the non-oxidative PPP (non-oxPPP) [29], thereby directly fueling nucleotide synthesis. In addition, the PPP in JEV-infected N2a cells showed phase-specific activation, characterized by an increase in the levels of ribulose-5-phosphate (Ru5P) and non-oxidative branch metabolites such as E4P and xylulose-5-phosphate (Xu5P) (Fig 2F). Notably, the abundance of serine, which potentially contribute to folate-mediated one-carbon metabolism to support nucleotide biosynthesis, was also upregulated (Fig 2G). This aligns with the pronounced accumulation of purine pathway intermediates, including IMP, AMP, and other purine nucleosides and bases (Fig 2H). These findings reveal that JEV significantly disrupts CCM networks associated with nucleotide biosynthesis in N2a cells.

### [U-^13^C] Glucose tracing reveals an enhancement of *de novo* nucleotide biosynthesis flux induced by JEV infection in neurons

**Fig 3:**
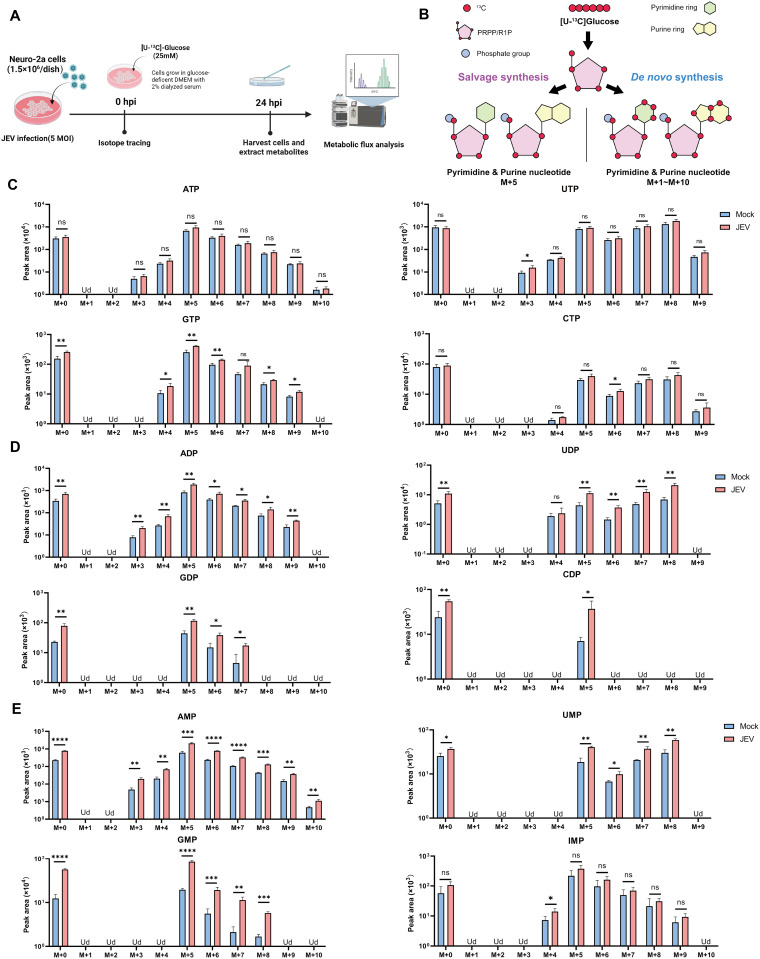
[U-¹³C] Glucose tracing reveals JEV-induced enhancement of *de novo* nucleotide biosynthesis flux in N2a cells. **(A)** Schematic of the experimental design for [U-¹³C] glucose tracing assay. N2a cells were infected with JEV at a MOI of 5, followed by incubation in glucose-deficient medium supplemented with dialyzed serum and [U-^13^C] glucose for 24 h. Created in BioRender. Gu, J. (2026) https://BioRender.com/f2uz4b3. **(B)** Pathways of *de novo* and salvage nucleotide biosynthesis showing the incorporation of glucose-derived carbon. The ribose sugar moiety derived from glucose contributes five ¹³C atoms (M + 5) to nucleotides, while the nucleobases incorporate ¹³C from various precursors (e.g., glycine, formate, aspartate, and glutamine) leading to intermediate (0 < M < 5) or fully labeled (M > 5) species when combined with labeled ribose. **(C–E)** Normalized peak areas of isotopologues for NTPs **(C)**, NDPs **(D)**, and NMPs **(E)** in mock- and JEV-infected N2a cells. Data are presented as mean ± s.e.m. (n = 3). Statistical significance was determined using two-tailed unpaired Student’s t-tests (*p < 0.05, **p < 0.01, ***p < 0.001, ****p < 0.0001).

To address the dynamic rewiring of CCM implied by our static metabolomic profiles, we conducted [U-^13^C] glucose tracing to track isotopic enrichment across metabolites of CCM in JEV-infected N2a cells at 24 hpi (Fig 3A). Following incubation with [U-^13^C] glucose, isotopic equilibration through multiple turns of TCA cycle resulted in diverse labeling patterns in cycle intermediates, as evidenced by the co-occurrence of M + 2 to M + 5 and fully labeled M + 6 isotopologues (S2A Fig). This dynamic carbon cycling reflects the iterative entry of ¹³C-acetyl-CoA and the anaplerotic replenishment of oxaloacetate pools. Our isotope tracing analysis revealed that JEV-infected N2a cells displayed elevated rates of [U-^13^C] glucose progressive incorporation into all detected TCA intermediates, including citrate, aconitate, α-ketoglutarate, succinate and malate (S2B Fig, S5 Table), confirming that JEV infection significantly enhanced TCA cycle flux in host cells. Interestingly, acetyl-CoA flux remained stable despite the elevated activity of TCA cycle driven by JEV, indicating JEV’s engagement of alternative pathways to sustain this increased TCA cycle activity. Concurrently, an increase in glycolytic metabolic flux was evidenced by the incorporation of ¹³C into M + 3 pyruvate and M + 3 lactate, suggesting a virally hijacked Warburg-like metabolic reprogramming (S2C Fig).

Nucleotide synthesis encompasses both *de novo* synthesis and the salvage synthesis pathway. In general, proliferating cells predominantly depend on *de novo* nucleotide biosynthesis to satisfy the heightened demand for DNA/RNA precursors during rapid proliferation [30,31]; whereas neurons primarily rely on nucleotide salvage pathways due to their higher levels of salvage enzymes like HPRT but lack key *de novo* synthesis machinery [22,32], making salvage pathways essential for maintaining nucleotide pools. To investigate whether the rapid replication of JEV influences the nucleotide biosynthesis pathway in neurons, we performed metabolic flux analysis by tracking the incorporation of [U-¹³C] glucose-derived carbon into nucleotide mono-, di-, and triphosphates, as well as 5-phosphoribosyl-1-pyrophosphate (PRPP) (Fig 3B). Pre-existing unlabeled nucleotide pools within cells contribute to the generation of M + 0 isotopologues, reflecting the incorporation of endogenous, non-¹³C-labeled precursors during nucleic acid turnover. Since the ribose moiety of nucleotides typically incorporates five ¹³C atoms from [U-^13^C] -glucose [30], M + 5 labeled nucleotides may originate from either *de novo* synthesis requiring PRPP-dependent assembly of small-molecule precursors, or salvage pathways involving attachment of a labeled ribose to pre-existing unlabeled nucleobases (Fig 3B) [33]. The ¹³C-labeling patterns of nucleobases illustrate the integration of carbon precursors from multiple pathways. Glycine, formate, glutamine, and aspartate collectively contribute to the progressive labeling, generating 0 < M < 5 intermediates initially, before reaching fully labeled states (M > 5 when combined with the labeled ribose moiety). Despite isotopic labeling for 24h, substantial unlabeled nucleotide fractions (M + 0) persisted in both the mock- and JEV-infected groups, indicating a continuous turnover of pre-existing nucleotide pools (Fig 3C–3E). In terms of nucleotide triphosphates (NTPs), GTP exhibited comprehensive upregulation across multiple isotopologues, while CTP and UTP demonstrated increases only in the M + 6 and M + 3 fractions, respectively (Fig 3C). Although the relative distribution of ATP isotopologues shifted, with M + 1 to M + 5 fractions increasing and M + 6 to M + 10 fractions decreasing (S2D Fig), the normalized peak area data showed no significant change in either individual isotopologue abundance following JEV infection (Fig 3C), indicating that JEV infection does not grossly alter ATP metabolic flux. However, JEV infection widely increased the levels of detected isotopologues of nucleotide diphosphates (NDPs) and nucleotide monophosphates (NMPs) compared to the mock-infected controls (Fig 3D and 3E). The increased dominant M + 5 fractions of these NMPs may indicate coordinated activation of both *de novo* synthesis and salvage pathways. In contrast, only the M + 4 fraction of IMP was elevated in JEV-infected cells (Fig 3E). Further analysis of the distributions of 0 < M < 5 and M > 5 fractions of these NMPs revealed a significant upregulation of both partially labeled (M + 1 to M + 4) and fully labeled (M + 6 to M + 10) NMP species during JEV infection in N2a cells (S2E Fig). This metabolic reprogramming was further corroborated by the elevated levels of M + 5 labeled PRPP, an essential gateway metabolite for *de novo* purine and pyrimidine synthesis (S2F Fig). The coordinated upregulation of PRPP and downstream labeled NMPs suggests that JEV strategically hijacks the host’s *de novo* nucleotide biosynthesis by augmenting PRPP supply through the PPP and accelerating the integration of carbon-nitrogen into nucleobases. Moreover, the limited detection of M + 1 to M + 4 nucleotide fractions and the high levels of M > 5 nucleotides observed in the results likely reflects the slower kinetics of ¹³C incorporation into nucleobases compared to ribose moieties (S2E Fig). Taken together, these findings demonstrate that JEV infection profoundly reprograms the CCM flux in N2a cells, with particularly striking alterations in nucleotide biosynthetic pathways. Our isotopic tracing data provide direct evidence that this rewiring of nucleotide biosynthesis is mediated, at least in part, by enhanced *de novo* nucleotide synthesis.

### JEV hijacks *de novo* purine synthesis for its replication in neurons

**Fig 4:**
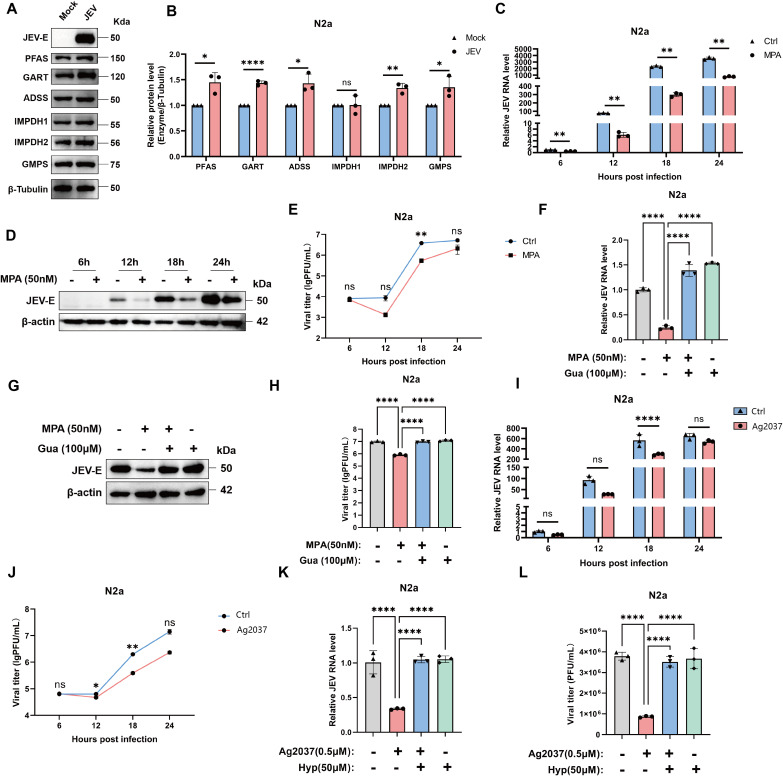
JEV hijacks *de novo* purine synthesis for its replication in neurons. **(A–B)** Impact of JEV infection on protein expression of key DNPB enzymes. N2a cells were infected or mock-infected with JEV at an MOI of 1. The expression of PFAS, GART, IMPDH1, IMPDH2, ADSS, GMPS, and β-Tubulin proteins were detected by immunoblotting, and representative blots from 3 independent experiments are presented **(A)**. Protein levels were quantified by using Image J software, normalized to β-Tubulin, and presented relative to the mock-infected control (n = 3) **(B)**. **(C–E)** Inhibitory effect of MPA on JEV replication. N2a cells were infected with JEV at an MOI of 5, followed by treatment with either 50 nM MPA or an equivalent volume of DMSO at 1.5 hpi. The levels of intracellular viral RNA **(C)** and E protein **(D)**, as well as the infectious virus production in the supernatant **(E)** at 6, 12, 18, and 24 hpi, were determined using qRT-PCR, immunoblotting, and plaque assay, respectively. **(F–H)** The effect of guanosine (Gua) treatment on MPA-mediated inhibition of JEV replication. N2a cells infected with JEV were treated with either MPA or DMSO, with or without addition of 100 μM Gua. The levels of intracellular viral RNA **(F)** and E protein **(G)**, as well as the production of infectious virus in the supernatant **(H)**, were determined using qRT-PCR, immunoblotting, and plaque assay, respectively, at 18 hpi. **(I–J)** Inhibitory effect of Ag2037 on JEV replication. N2a cells were infected with JEV at an MOI of 5, followed by treatment with either 0.5 μM Ag2037 or an equivalent volume of DMSO at 1.5 hpi. The levels of intracellular viral RNA **(I)** and viral titers in the supernatant **(J)** were determined at 6, 12, 18, and 24 hpi. **(K–L)** The effect of hypoxanthine (Hyp) treatment on Ag2037-mediated inhibition of JEV replication. N2a cells infected with JEV were treated with either Ag2037 or DMSO, with or without addition of Hyp at 1.5 hpi. The levels of intracellular viral RNA **(K)** and viral titers in the supernatant **(L)** were measured at 18 hpi. Data are presented as mean ± s.e.m. from 3 independent biological replicates (n = 3). Significance was determined using two-tailed unpaired Student’s t-test **(B)**, one-way **(F, H, K and L)** or two-way ANOVA **(C, E, I and J)** (*p < 0.05, **p < 0.01, ***p < 0.001; ****p < 0.0001; ns, not significant).

Our metabolic analysis suggested that *de novo* nucleotide pathway was activated in N2a cells during JEV infection. Given that the genome of JEV P3 strain exhibits a purine-biased nucleotide composition (A: 27.61%, G: 28.48%, C: 22.96%, U: 20.95%), with purines (A + G, 56.09%) significantly outnumbering pyrimidines (C + U, 43.91%) (S3A and S3B Fig), this likely reflects a higher metabolic demand for purine precursors during viral replication. Consequently, we prioritized the investigation of JEV's manipulation of host purine biosynthesis over pyrimidine pathways. DNPB requires ten enzymatic steps to synthesize IMP from PRPP. Subsequently, IMP undergoes divergent metabolic pathways catalyzed by IMPDH/GMPS or ADSS/ADSL, leading to the production of GMP and AMP, respectively. To deepen our understanding of how JEV modulates *de novo* purine biosynthesis (DNPB), we measured the mRNA levels of relevant enzymes in JEV infected N2a cells and primary neurons. The results revealed a significant transcriptional upregulation of all core components of the DNPB pathway in N2a cells infected with 5 MOI of JEV at 24 and 36 hpi (S4A Fig), or 1 MOI of JEV at 48 hpi (S4B Fig). A similar transcriptional upregulation of these enzymes was also observed in mouse primary neurons and TM4 cells (a mouse Sertoli cell line) (S4C and S4D Fig). Subsequently, we assessed the corresponding changes at the protein level using Western blot analysis. Consistent with the qPCR results, the protein levels of PFAS, GART, IMPDH2, ADSS, and GMPS were significantly upregulated at 48 h post infection of JEV at 1 MOI (Fig 4A and 4B). Collectively, these results demonstrate a widespread upregulation of *de novo* purine biosynthetic enzymes in JEV-infected neurons.

Additionally, we analyzed our previous scRNA-seq datasets from JEV-infected mouse brains [34], focusing specifically on transcriptional expression of DNPB enzymes in neuronal populations. Based on the levels of viral gene expression, we classified all neuronal cells into five distinct groups: the mock-infected group (Mock), JEV-exposed but viral genome-negative neurons (JEV-N, JEV expression = 0), viral genome-low neurons (JEV-L, log2[JEV expression] < 3), viral genome-medium neurons (JEV-M, 3 ≤ log2[JEV expression] ≤ 8), and viral genome-high neurons (JEV-H, log2[JEV expression] > 8), establishing a comprehensive gradient model to investigate neuronal responses across the entire spectrum of infection intensity, ranging from viral exposure to high viral burden. As JEV infection progressed, we observed a striking divergence in the transcriptional dynamics of DNPB enzymes (S4E Fig). The mRNA levels of PAICS, PFAS, GMPS, and ADSS showed a sustained decrease with increasing viral burden. Notably, these enzymes catalyze ATP- and GTP-consuming steps, suggesting that JEV infection suppresses the energy-intensive branches of DNPB to reduce host metabolic expenditure. In contrast, the virus appeared to selectively hijack the pathway’s rate-limiting nodes PPAT and IMPDH1 transcripts were downregulated at low infection levels but markedly upregulated in heavily infected neurons, highlighting a biphasic regulation pattern. Given the isoform-specific expression of IMPDH1 and IMPDH2 in different tissues, our data further suggest a possible isoform switch during infection, with IMPDH1 induction compensating for the decline of IMPDH2 to sustain GTP availability under high viral load. Interestingly, in low and medium level infected neurons, GART, ADSL, IMPDH2, and ATIC were transiently upregulated, peaking in the low-infection groups before declining at higher viral burdens. Thus, our single-cell transcriptomic profiling of infected mouse brain neurons reveals a more dynamic and refined transcriptional landscape that is induced by JEV infection. This landscape includes the sustained downregulation of several ATP- or GTP-consuming enzymes alongside the biphasic upregulation of key rate-limiting enzymes such as PPAT and IMPDH1 in heavily infected neurons.

To investigate the role of DNPB in JEV replication, we next pharmacologically inhibited IMPDH, the rate-limiting enzyme in this pathway, using MPA. The results demonstrated that treatment with 50 nM MPA, which maintained over 90% cell viability (S5A Fig), significantly suppressed JEV RNA replication (Fig 4C), reduced viral E protein expression (Fig 4D), and decreased viral titers (Fig 4E) compared to the DMSO-treated group. To confirm pathway specificity, we rescued MPA-mediated inhibition by supplementing guanosine (Gua), the downstream product of IMPDH. Remarkably, addition of 100 μM Gua fully restored viral RNA levels (Fig 4F), E protein expression (Fig 4G), and infectious particle production (Fig 4H). These findings establish the critical dependence of JEV replication on *de novo* purine biosynthesis. To systematically investigate the impact of DNPB upstream pathways on JEV replication, we treated the N2a cell with 0.5 μM Ag2037 (S5B Fig), an inhibitor of the GART enzyme. Similar results were obtained with Ag2037 inhibition of JEV replication (Fig 4I and 4J). Furthermore, the antiviral effect of Ag2037 could be completely rescued by exogenous supplementation with hypoxanthine, a critical downstream metabolite of the DNPB pathway (Fig 4K and 4L). Collectively, these findings indicate that JEV hijacks *de novo* purine synthesis for its replication in host neuronal cells.

### JEV reprograms the pentose phosphate pathway to fuel nucleotide biosynthesis via non-oxidative branch dependency in neurons

**Fig 5:**
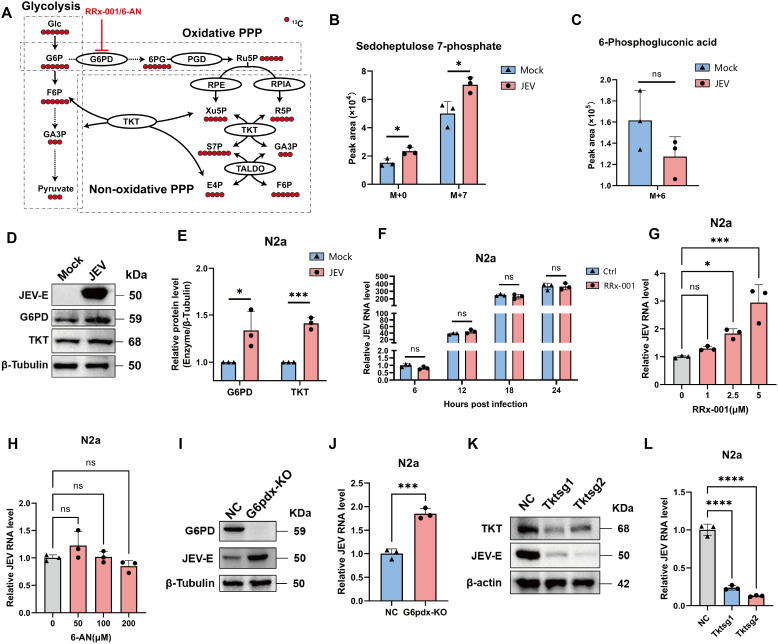
JEV reprograms the PPP to fuel nucleotide biosynthesis through non-oxidative branch dependency in neurons. **(A)** Schematic of the pentose phosphate pathway, highlighting the oxidative branch and the non-oxidative branch. **(B–C)** Metabolic flux analysis using [U-¹³C] glucose tracing. The flux in the non-oxidative branch intermediate sedoheptulose-7-phosphate (M + 7) **(B)** and the oxidative branch intermediate 6-phosphogluconate (M + 6) **(C)** were presented. **(D–E)** Expression of the key PPP enzymes G6PD and TKT in N2a cells infected or mock-infected with JEV was detected using immunoblotting at 36 hpi, and the representative blots were presented **(D)**. The relative expression levels of these proteins were calculated **(E)**. **(F–J)** Effect of the oxidative PPP branch inhibitors on JEV replication. N2a cells were infected with JEV at an MOI of 5, followed by treatment of 250 nM RRX-001 at 1.5 hpi. The relative viral RNA levels at indicated time points were measured **(F)**. Additionally, increasing concentrations of RRX-001 **(G)** or 6-AN **(H)** were used to treat the JEV-infected N2a cells, and the viral RNA levels were determined. **(I–J)** The regulatory role of G6PD in JEV replication in N2a cells. The *G6pdx* knockout (KO) N2a cell line and the control cells (NC) were infected with JEV at an MOI of 5. The expression of G6PD and JEV E proteins **(I)** and the viral RNA levels **(J)** were assessed at 24 hpi. **(K–L)** The regulatory role of TKT in JEV replication in N2a cells. *Tkt*-deficient (sg1 and sg2) and negative control (NC) N2a cells were infected with JEV at an MOI of 5. The expression of TKT and JEV E proteins **(K)** and the viral RNA levels were determined **(L)**. Data are presented as mean ± s.e.m. from 3 independent biological replicates (n = 3). Significance was determined by two-tailed unpaired Student’s t-test (**B-C, E, J and L**), one-way ANOVA **(G-H)**, or two-way ANOVA **(F)** (*p < 0.05, **p < 0.01, ***p < 0.001, ****p < 0.0001; ns, not significant).

PPP serves as the primary source of ribose-5-phosphate (R5P), an essential precursor for PRPP synthesis required by DNPB. The PPP comprises both oxidative (generating NADPH and Ru5P) and non-oxidative (producing R5P) branches (Fig 5A) [35]. Metabolic flux analysis revealed that JEV infection induces a pronounced divergence between the oxidative and non-oxidative branches of the PPP. Specifically, M + 7 labeled sedoheptulose-7-phosphate (S7P) in the non-oxidative branch exhibited increased flux (Fig 5B), whereas intermediates of the oxidative branch, such as M + 6 6-phosphogluconic acid, remained unchanged (Fig 5C). To characterize the modulation of PPP enzyme expression in neurons by JEV infection, we first conducted qRT-PCR on JEV-infected N2a cells, primary neurons and TM4 cells, which confirmed a consistent upregulation pattern for enzymes in the PPP (S4F-S4H Fig). Subsequently, we assessed the protein expression levels of two key PPP enzymes, G6PD and TKT. Western blot analysis revealed that both enzymes were upregulated following JEV infection in N2a cells (Fig 5D and 5E). The scRNA-seq analysis also demonstrated that neurons from JEV-infected mice exhibited significantly elevated transcription of PPP-related enzymes in neurons with low and medium viral burden neurons compared to the Mock group (S4I Fig).

To investigate whether the PPP contribute to JEV replication in neurons, we employed G6PD inhibitors, RRx-001 (S5C Fig) and 6-AN (S5D Fig), to suppress the oxidative branch during JEV infection in N2a cells. However, treatment with these inhibitors failed to suppress viral replication (Fig 5F–5H), and a high dose of RRx-001 even paradoxically enhanced JEV RNA accumulation (Fig 5G). This phenotype was further confirmed in *G6pdx* knockout (KO) cells, where viral RNA and protein expression also significantly increased compared to negative control (NC) N2a cells (Fig 5I and 5J). We subsequently assessed the role of the non-oxidative PPP in JEV replication. We found that deletion of the non-oxidative branch enzyme gene *Tkt* resulted in a significant reduction of JEV RNA and protein levels (Fig 5K and 5L), confirming its critical role in sustaining infection. These results highlighted a critical dependency of JEV replication on the non-oxidative branch of the PPP.

### MTHFD2-dependent one-carbon metabolism sustains JEV replication in neurons

**Fig 6:**
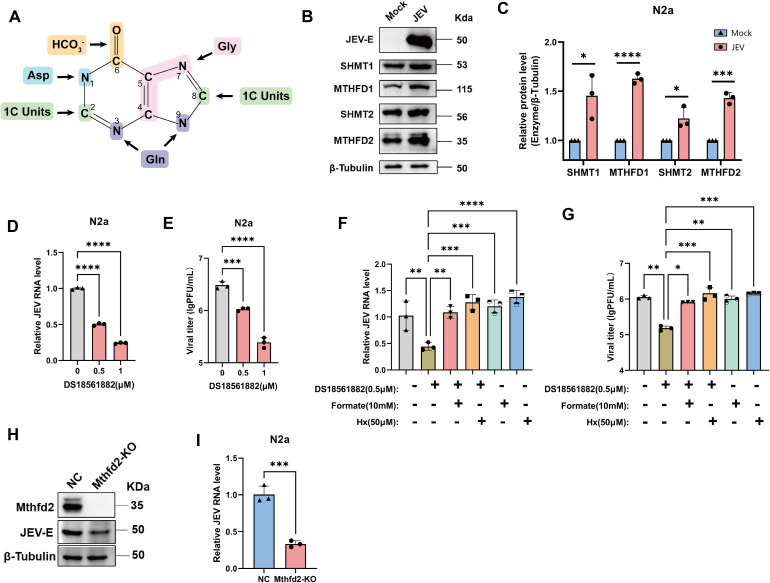
MTHFD2-dependent mitochondrial one-carbon metabolism is essential for JEV replication in neurons. **(A)** Schematic diagram illustrating the metabolic origins of purine ring atoms, highlighting the contribution of key metabolites. One-carbon (1C) units are specifically required for purine ring assembly at the C2 and C8 positions. **(B–C)** Expression of key 1C metabolic enzymes in JEV-infected N2a cells. N2a cells were infected or mock-infected with JEV at an MOI of 5. The indicated enzymes were detected by using Western blotting at 36 hpi **(B)**, and the relative protein levels were calculated **(C)**. Representative blots from 3 independent experiments are presented. (D–E) Effect of MTHFD2 inhibitor on JEV replication. N2a cells were infected with JEV at an MOI of 5, followed by treatment with increasing concentrations of DS18561882 at 1.5 hpi. The viral RNA levels (D) and titers (E) were assessed at 18 hpi. (F–G) Effect of sodium formate and hypoxanthine on DS18561882-mediated inhibition of JEV replication. JEV-infected N2a cells were treated with DS18561882, supplemented with or without sodium formate or hypoxanthine. The viral RNA levels (F) and titers (G) were determined at 18 hpi. (H–I) Role of MTHFD2 on JEV replication in N2a cells. *Mthfd2* KO N2a cells line and the negative control cells were infected with JEV at an MOI of 5. The viral RNA accumulation (H) and E protein expression (I) were measured. Data are presented as mean ± s.e.m. from 3 independent biological replicates (n = 3). Significance was determined by two-tailed unpaired Student’s t-test (C and **I) or one-way ANOVA (D-G)**. (*p < 0.05, **p < 0.01, ***p < 0.001, ****p < 0.0001; ns, not significant).

The DNPB pathway requires 1C units as essential building blocks for purine ring assembly (Fig 6A) [36]. Our integrated bulk RNA-seq and metabolomic analysis of JEV-infected mouse brains revealed marked transcriptional upregulation of key one-carbon metabolic enzymes and significant depletion of 10-formyl-THF (Fig 1D and 1E), implying that the virus hijacks 1C units through remodeling host one-carbon metabolism. Therefore, we speculated that folate-driven one-carbon metabolism supplies the necessary 1C units for purine synthesis, and the dynamic regulation of its enzymatic machinery may serve as a critical node for JEV replication. As expected, the upregulation of both mRNA and protein levels of key one-carbon metabolic enzymes was validated in JEV-infected N2a cells using qRT-PCR (S4J Fig) and immunoblotting (Fig 6B and 6C), respectively. Except *Aldh1l1*, all detected enzymes increased significantly in their transcriptional levels in JEV-infected primary neurons (S4K Fig). Consistent with these neuronal findings, MTHFD1 and MTHFD2 mRNA expression was also upregulated in TM4 cells (S4L Fig). Intriguingly, the scRNA-seq analysis at single-neuron resolution revealed a compartmentalized shift in enzyme expression: mildly infected neurons (JEV-L) predominantly expressed cytosolic enzymes such as SHMT1 and MTHFD1, whereas severely infected neurons (JEV-H) exhibited elevated expression of mitochondrial enzymes like MTHFD2 and SHMT2 (S4M Fig).

To investigate the impact of 1C metabolism on JEV replication in neurons, we treated N2a cells with DS18561882 (S5E Fig), a selective inhibitor of MTHFD2, to obstruct the 1C metabolic pathway during JEV infection. Our results demonstrated that DS18561882 effectively inhibited JEV RNA replication and significantly reduced viral titers in a dose-dependent manner (Fig 6D and 6E). Notably, the supplementation of sodium formate or hypoxanthine remarkably rescued JEV replication efficiency (Fig 6F and 6G), confirming that DS18561882 specifically suppresses viral replication through MTHFD2 inhibition and that JEV replication depends strictly on functional one-carbon metabolism to furnish essential precursors for purine nucleotide synthesis. To further validate the essential role of MTHFD2 in JEV replication, we generated a *Mthfd2* KO N2a cell line. The *Mthfd2* deficiency significantly suppressed both JEV RNA and protein synthesis (Fig 6H and 6I). These findings collectively establish mitochondrial 1C metabolism, particularly MTHFD2-mediated folate cycling, as a critical metabolic vulnerability for JEV replication in neuronal cells.

### Ag2037 treatment reduces viral loads and attenuates neuroinflammation in mouse brains during JEV infection

**Fig 7:**
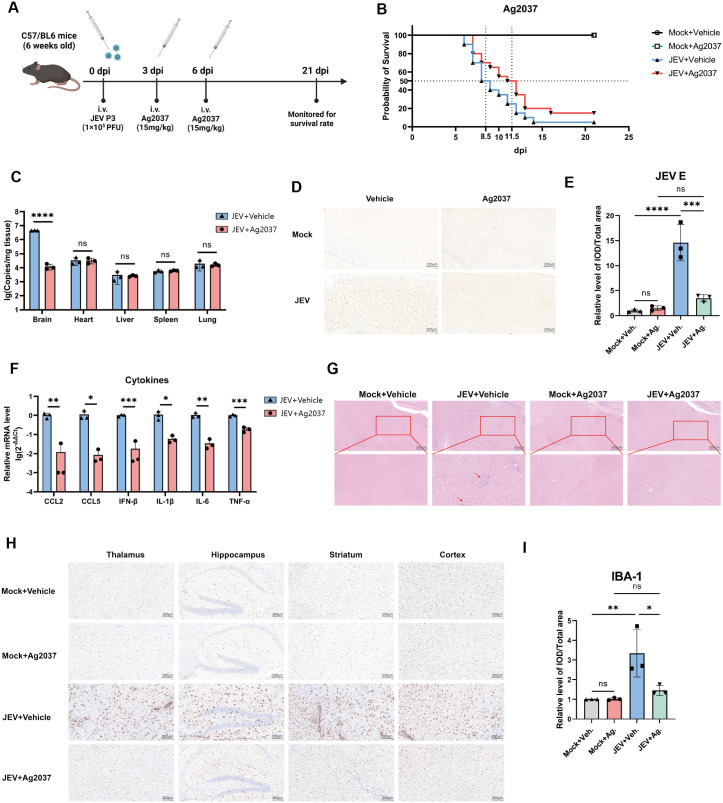
Ag2037 treatment reduces viral load and attenuates neuroinflammation in JEV-infected mice. Six-week-old C57/BL6 mice were intravenously infected or mock-infected with 10^5^ PFU JEV, followed by i.v. injected with 15mg/kg Ag2037 at 3 and 6 dpi **(A)**. Created in BioRender. Gu, J. (2026) https://BioRender.com/f2uz4b3. The survivals of treated mice were monitored until 21 dpi (n = 20 mice/group) **(B)**. The JEV RNA copies and the expression of E protein in the brains were determined using RT-qPCR (n = 3 mice/group) (C) and immunohistochemical staining (D) at 6 dpi. The integrated optical density (IOD) of E protein signals was calculated with 3 sections for each mouse (n = 3 mice/group) using Image-Pro Plus 6.0 software **(E)**. Additionally, the mRNA levels of type I interferon and pro-inflammatory cytokines (IFN-β, TNF-α, CCL-2, CCL-5, IL-6, IL-1β) in brain tissues were measured using RT-qPCR at 6 dpi (n = 3 mice/group) **(F)**. Moreover, the brain sections were subjected to H&E staining **(G)** and immunohistochemistry staining of IBA-1 **(H)**, with the IOD of IBA signals being calculated **(I)** (n = 3 mice/group). Data are presented as mean ± s.e.m.. Statistical significance was determined using log-rank test **(B)**, two-tailed unpaired Student’s t-test **(C** and **F)**, or one-way ANOVA **(E** and **I)** (*p < 0.05, **p < 0.01, ***p < 0.001, ****p<0.0001).

To further assess the role of DNPB in JEV replication *in vivo*, we employed the GART inhibitor Ag2037 in a JEV-infected mouse model. To minimize its inhibitory effects on DNPB in peripheral tissues, mice that either infected or mock-infected with JEV were administered with Ag2037 intravenously at 3 and 6 dpi (Fig 7A), considering that CNS invasion of JEV has been reported to occur at 3 dpi [37]. Our findings revealed that JEV-infected mice treated with Ag2037 exhibited milder symptoms and improved survival outcomes compared to the vehicle-treated group, as evidenced by an extended time-to-death (median survival of 11.5 days vs 8.5 days) and a reduced mortality rate (Fig 7B). Consistent with these survival benefits, Ag2037 significantly suppressed viral RNA accumulation and E protein expression in the mouse brain at 6 dpi as demonstrated by RT-PCR and immunohistochemistry analysis (Fig 7C–7E). As anticipated, a significant reduction in JEV viral RNA copies was observed exclusively in the brain tissues of Ag2037-treated mice, rather than in other peripheral tissues (Fig 7C), indicating that the effect of Ag2037 was limited to the central nervous system under the conditions of this study.

Considering the reduced viral loads observed in the mouse brain, which may influence the neuroinflammation caused by JEV, we next investigated the effect of Ag2037 treatment on the inflammatory response in the brains of JEV-infected mice. As shown in the results, Ag2037 treatment significantly downregulated the mRNA levels of type I interferon and pro-inflammatory cytokines, including IFN-β, TNF-α, CCL-2, CCL-5, IL-6, and IL-1β, compared to vehicle treatment (Fig 7F). Histopathological examination of brain sections via H&E staining further revealed that vehicle-treated brains displayed prominent perivascular inflammatory cuffing, indicative of lymphocyte infiltration and blood-brain barrier disruption, whereas Ag2037-treated tissues displayed alleviative pathological changes (Fig 7G). To dissect the cellular mechanisms underlying this attenuated inflammatory response, we assessed microglial activation through IBA-1 immunohistochemistry staining. Ag2037 treatment significantly suppressed microglial hyperactivation, as evidenced by a reduction in IBA-1 integrated optical density (IOD) compared to vehicle-treated controls (Fig 7H and 7I). Taken together, our data demonstrate that the administration of Ag2037 inhibits JEV replication in the CNS and attenuated the neuroinflammatory response, highlighting its therapeutic efficacy in the context of Japanese encephalitis.

## Discussion

JEV remains a significant global health threat, particularly in Asia and Australia [38], yet no specific antiviral therapy has been approved for clinical use [39]. Several reports have suggested that the development of novel antiviral strategies, such as host-targeted metabolic inhibitors, could provide a breakthrough for anti-viral treatment [40–42]. In this study, we conducted a metabolomic analysis in both mouse brain and N2a cells during JEV infection, and identified DNPB, PPP, and 1C metabolism as host-derived metabolic vulnerabilities in JEV-infected neurons, which could potentially serve as therapeutic targets for JEV infection.

Our data demonstrate that JEV infection enhances glycolytic flux in N2a cells, evidenced by increased M + 3 pyruvate and lactate labeling, a metabolic signature reminiscent of the Warburg effect observed in several viral infections [43–45]. These findings are consistent with recent work demonstrating HIF-1α–mediated enhancement of glycolysis in JEV-infected N2a cells [46]. However, their analysis did not resolve intermediate metabolites or downstream metabolic pathways. In contrast, our targeted metabolomics and [U-¹³C] glucose tracing provided a systems-level view of central carbon metabolism, capturing dynamic carbon flux through glycolysis and its integration with downstream pathways. Interestingly, despite increased glycolytic and TCA cycle fluxes upon viral infection, neither the abundance nor the ¹³C enrichment of acetyl‑CoA was elevated. This suggests that acetyl‑CoA is maintained at a steady‑state level with rapid turnover, likely due to a balance between increased production and enhanced consumption. Additionally, the TCA cycle can be fueled by multiple upstream pathways, and the increased flux observed here may also reflect coordinated metabolic inputs. This dual enhancement of glycolysis and TCA flux parallels metabolic strategies employed by human cytomegalovirus [47] and African swine fever virus [48], and likely supports both energy production and biosynthetic precursor supply for viral replication.

Nucleotides are essential building blocks for viral RNA synthesis. In this study, we found that nucleotide metabolism emerged as a significantly enriched pathway in JEV-infected mouse brains, with pronounced upregulation of orotic acid and UMP, providing direct evidence of enhanced *de novo* nucleotide synthesis in brains. The marked accumulation of xanthine and urate in JEV-infected mouse brains, coupled with the transcriptional induction of XDH, indicates enhanced purine catabolism *in vivo*. Under normal physiological conditions, the adult brain exhibits minimal xanthine oxidoreductase activity and maintains low urate levels, with hypoxanthine serving as the major purine degradation product that can be salvaged [49]. Moreover, the conversion of hypoxanthine to xanthine and urate is largely irreversible, and these oxidized products are typically exported from cells, thereby limiting their reutilization and effectively reducing the purine pool. This catabolic drain may increase the reliance of infected neurons on *de novo* purine biosynthesis.

A recent untargeted metabolomics study also reported perturbations in purine metabolism in JEV-infected mouse brains [50]. However, their analysis was limited to correlative changes in metabolite abundance without addressing the underlying mechanisms or metabolic flux. In contrast, our study provides functional and flux-level evidence that these alterations reflect reprogramming of nucleotide biosynthesis in infected neurons. Our isotopic tracing experiments revealed that JEV infection enhances both *de novo* purine and pyrimidine biosynthesis flux, as evidenced by elevated M > 5 labeled NMP and NDP. Importantly, despite the enhanced *de novo* synthesis flux, steady‑state pool sizes of ATP and GTP remained largely unchanged, suggesting that increased nucleotide production is tightly coupled to the immediate demands of viral RNA replication. Considering the purine‑biased nucleotide composition of the JEV genome, we hypothesize that viral replication critically depends on host purine biosynthesis. Pharmacological inhibition of DNPB using MPA and Ag2037 significantly suppressed JEV replication in N2a cells, indicating that DNPB is essential for JEV life cycle. Exogenous purine supplementation restored JEV replication efficiency under DNPB inhibition, demonstrating the virus’s flexible utilization of salvage pathway. Although this study demonstrates the reliance of JEV on *de novo* purine biosynthesis, the role of the salvage pathway, the primary source of nucleotides in the CNS, remains unclear. Given its importance in maintaining neuronal nucleotide pools, it may also contribute to JEV replication.

The PPP supports viral replication by providing ribose‑5‑phosphate for nucleotide synthesis and NADPH for redox balance and fatty acid biosynthesis [51]. While several viruses upregulate the oxidative PPP for antioxidant defense and nucleotide production [52–54] and a previous report specifically proposed that JEV relies on this oxidative branch [55], our data reveal a distinct dependency in JEV‑infected neurons. Inhibition or knockout of G6PD unexpectedly enhanced JEV replication, whereas TKT knockdown suppressed it. These findings indicate that JEV preferentially relies on the non‑oxidative PPP branch for ribose‑5‑phosphate supply, while the oxidative branch may exert antiviral effects through NADPH‑mediated redox regulation. We also note that our [U‑¹³C] glucose tracing approach cannot resolve the relative contributions of each branch for ribose‑5‑phosphate production, future studies using [1,2‑¹³C] glucose will be required to quantify this partitioning.

Emerging evidence indicates that diverse viruses hijack host 1C metabolism to support purine synthesis [41,54,56]. In JEV‑infected brains, we observed depletion of 10‑formyl‑THF, suggesting heightened 1C unit consumption. Pharmacological inhibition of MTHFD2 suppressed viral replication, rescued by formate or hypoxanthine, demonstrating that JEV replication requires MTHFD2‑derived 1C units for *de novo* purine biosynthesis.

Single-cell transcriptomic data from mouse brain neurons revealed a more dynamic and refined pattern of virus–host metabolic interactions during JEV infection, likely more representative of *in vivo* conditions than bulk brain transcriptomes or the N2a models. Notably, sustained downregulation of ATP/GTP-consuming enzymes and biphasic upregulation of PPAT and IMPDH1 were observed in infected neurons. However, whether these transcriptional changes translate into alterations in protein abundance, enzyme activity, and metabolic flux remains unclear. Future studies employing single-cell proteomics or metabolic flux analyses in infected brain tissue will be required to define the functional significance of these transcriptional signatures. Nevertheless, our work provides a conceptual framework for understanding neuronal metabolic responses to neurotropic viral infection and highlights potential regulatory nodes for further investigation. Through our integrated analysis of murine brain transcriptomes, neuronal single-cell RNA sequencing, and quantitative PCR validation, we revealed that JEV infection broadly induces the transcriptional upregulation of metabolic enzymes across the DNPB, PPP, and 1C metabolism pathways. Additionally, JEV infection also upregulated several DNPB, PPP, and 1C metabolic enzyme transcripts in TM4 cells, a non‑neuronal cell line permissive to JEV infection, indicating that this transcriptional induction is not strictly neuron‑specific. However, the upstream transcriptional regulators responsible for mediating this metabolic reprogramming remain to be elucidated through systematic investigation.

Given the efficient antiviral capability of DNPB inhibitors against JEV *in vitro*, we utilized a GART inhibitor Ag2037 to validate the role of DNPB *in vivo*. Notably, previous work by Sebastian et al. demonstrated antiviral activity of the IMPDH inhibitor MPA against JEV [57], a finding consistent with our results *in vitro*. However, IMPDH inhibition affects both *de novo* and salvage pathways, whereas GART targeting enables selective blockade of *de novo* purine biosynthesis, which is particularly relevant in the CNS where salvage predominates. Considering the absence of published data on the BBB permeability of Ag2037, drug administration was timed to coincide with JEV-induced BBB disruption beginning at 3 days post intravenous infection [37]. This approach minimizes potential peripheral antiviral effects of Ag2037 and focuses evaluation on CNS infection. Ag2037 treatment significantly attenuated early neuropathology and delayed mortality onset, though overall survival improved only modestly. The limited survival benefit may be attributed to the establishment of viral replication in CNS prior to BBB disruption [58–60]. Therefore, delayed pharmacological accessibility likely narrows the therapeutic window, limiting effective viral clearance and permitting sustained neuroinflammation driven by pre-existing infection. Additionally, infected neurons may sustain nucleotide pools via salvage pathways or nucleic acid catabolism. Consequently, combined inhibition of both *de novo* and salvage nucleotide biosynthesis pathways represents a promising strategy to deplete both endogenous and exogenous nucleotide sources and enhance antiviral efficacy [14].

**Fig 8:**
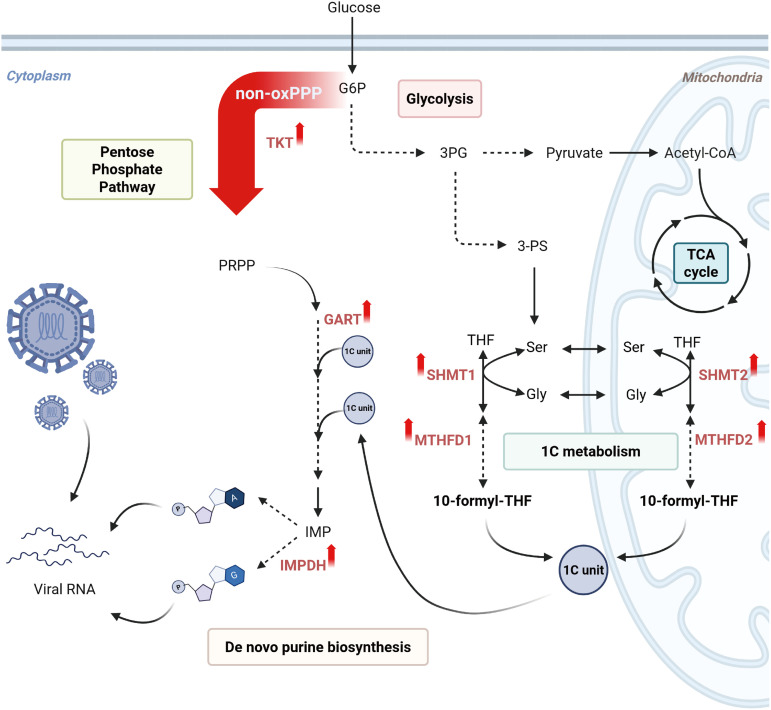
Schematic representation of JEV-induced reprogramming of central carbon and nucleotide metabolism to facilitate viral replication in neurons. JEV infection enhances glycolysis, PPP, and *de novo* nucleotide synthesis, as evidenced by significant upregulation of relative metabolites and enzymes. JEV replication exhibits a strong reliance on *de novo* purine biosynthesis, one-carbon metabolism, and the non-oxidative branch of the PPP, which together supply essential precursors for viral RNA synthesis, which reveals the metabolic vulnerabilities for JEV replication and highlights nucleotide metabolism as a promising target for antiviral therapy. Created in BioRender. Gu, J. (2026) https://BioRender.com/0c7iaiz.

In summary, our study reveals that JEV reprograms host CCM by enhancing glycolysis, the TCA cycle, and the PPP in neurons. JEV infection dramatically alters host nucleotide metabolism in both the brains and neuronal models, as evidence suggesting that these changes are primary driven by the upregulation of *de novo* nucleotide synthesis. The genomic purine bias of JEV critically necessitates host purine synthesis, and JEV replication relies on non-oxPPP and MTHFD2-driven 1C metabolism, which supply ribose-5-phosphate and formyl groups essential for nucleotide synthesis (Fig 8). Selective targeting of key enzymes governing these metabolic nodes disrupts viral replication *in vitro*, and inhibition of GART similarly suppresses viral replication *in vivo*, suggesting that host-directed metabolic interventions could serve as a strategic antiviral approach. These findings advance our understanding of the metabolic interactions between viruses and hosts and unveil novel therapeutic strategies for infectious diseases caused by JEV and other neurotropic viruses.

## Materials and methods

### Ethics statement

Female C57BL/6 mice were obtained from the Animal Center of Huazhong Agricultural University, Wuhan, China. All mouse experiments were conducted in accordance with the approved protocols of the Animal Care and Ethics Committee of Huazhong Agricultural University, under the reference number HZAUMO-2025–0082. The protocols followed the guidelines outlined in the Guide for the Care and Use of Laboratory Animals.

### Cells, viruses, and viral titration

Primary neuron cultures were prepared from the cerebral cortices of C57BL6J mice at embryonic day 16, as previously reported [61]. Briefly, the isolated primary cells were seeded on poly-lysine-coated (100 mg/mL) plates at a density of 6 × 10^5^ cells per milliliter in Dulbecco’s modified Eagle’s medium (DMEM) supplemented with 5% fetal bovine serum (FBS). At 6 hours (h) post-seeding, the culture medium was replaced with neurobasal medium supplemented with 2% B-27. The cells were used for subsequent experiments after culture for 7 days with half of the medium replaced by fresh medium every 3.5 days.

N2a cells (mouse neuroblastoma cell line), HEK-293T cells (human embryonic kidney cell line), TM4 cells (mouse Sertoli cell line) and BHK-21 cells (baby hamster kidney cell line) were cultured and maintained in DMEM supplemented with 10% FBS, 100 U/ml penicillin, and 100 µg/ml streptomycin sulfate.

The JEV P3 strain was propagated in the brains of suckling mice and BHK-21 cells for *in vivo* and *in vitro* experiments, respectively. The viral titer was determined using a plaque assay on BHK-21 cells. In brief, supernatants from infected cells were harvested and serially diluted 10-fold in DMEM, followed by inoculation onto BHK-21 cells. After incubation at 37 °C for 1.5 h, the supernatants were discarded, and the cells were incubated for 4 days in DMEM containing 2% FBS and 1.5% sodium carboxymethyl cellulose at 37 °C. The cells were then fixed with 10% formaldehyde overnight and stained with crystal violet solution for 2 h. The final viral titers were calculated based on the numbers of visible plaques counted.

### Reagents and antibodies

Mycophenolic acid (MPA, HY-108325), Ag2037 (HY-14530), DS18561882 (HY-130251), RRx-001 (HY-16438) and 6-Aminonicotinamide (6-AN, HY-101966) were purchased from MedChem Express. Sodium formate (71539), uridine (U3003), guanine (G6264), and hypoxanthine (H9636) were obtained from SigmaAldrich as exogenous 1C units and nucleotides. [U-^13^C] glucose was purchased from Cambridge Isotope Laboratories, Inc. (CLM-1396-1). Mouse anti-JEV-E monoclonal antibody (mAb) was prepared in our laboratory. Mouse anti-β-Actin mAb (HRP-66009), Rabbit anti-IMPDH1 polyclonal antibody (pAb, 22092-1-AP), anti-PFAS pAb (24716-1-AP), anti-TKT pAb (11039-1-AP), and anti-SHMT1 pAb (30192-1-AP) were purchased from Proteintech. Rabbit anti-GART pAb (A3876), anti-ADSS mAb (A20945), anti-IMPDH2 mAb (A9208), anti-GMPS pAb (A6606), anti-G6PD pAb (A13983), anti-MTHFD1 pAb (A8661), anti-SHMT2 pAb (A1215), anti-MTHFD2 mAb (A22653), and anti-β-Tubulin mAb (A12289) were purchased from ABclonal Technology. HRP conjugated goat anti-mouse IgG (BA1051) and anti-rabbit IgG (BA1055) were purchased from Boster. All commercial antibodies were used at concentrations recommended by the manufacturers.

### RNA extraction and quantitative reverse transcription-PCR (qRT-PCR)

Total RNA was extracted from treated samples using TRIzol reagent (Magen), according to the manufacturer’s instructions. Subsequently, 1 µg of RNA was used to synthesize cDNA with the ABScript II cDNA First-Strand Synthesis Kit (ABclonal Technology). Quantitative real-time PCR was performed using the 2 × University SYBR Green Fast qPCR Mix (ABclonal Technology) and a ViiA 7 Real-time PCR System (Applied Biosystems). The relative expression levels of the targeted mRNA were normalized to that of endogenous control β-actin within each sample, employing the 2^−ΔΔCt^ method. To determine the RNA copies of JEV in mouse brains, Taqman qRT-PCR was performed with the primers JEV-E-F, JEV-E-R, and the corresponding probe. A standard curve was generated with a 10-fold serial dilution of the plasmid encoding the JEV E gene. All primers and probes utilized are listed in S1 Table.

### Western blotting

Cells were lysed in RIPA buffer (Beyotime, P0013) supplemented with protease inhibitors (TargetMOI, C0001). Protein concentrations were determined using the Enhanced BCA Protein Assay Kit (Beyotime, P0010S). Equal amounts of protein were separated by SDS-PAGE and transferred onto a polyvinylidene fluoride (PVDF) membrane (Millipore) using a Mini Trans-Blot Cell (Bio-Rad). The membranes were blocked by incubation in TBS-T containing 2% BSA for 2 h, followed by incubation with the relevant primary antibodies at 4°C overnight. After washing three times with TBS-T, the membranes were then incubated with appropriate secondary antibodies at room temperature for 45 min and visualized using enhanced chemiluminescent (ECL) reagent (Tanon, China).

### Gene editing

All sgRNA sequences for gene editing were designed using CRISPOR [62]. The sgRNA oligos were annealed and subsequently cloned into the sgRNA/Cas9 expression vector (LentiCRISPRv2). A total of 800 ng of the LentiCRISPRv2 vector, 800 ng of the packaging plasmid pSPAX2, and 400 ng of the pMD2.G were co-transfected into HEK 293T cells using FuGENE HD Transfection Reagent (Promega, E2311), following the manufacturer's protocol. At 48 h post-transfection, viral supernatants were harvested and added to 4 × 10⁵ N2a cells. For *Tkt* gene silencing in N2a cells, the transduced cells were treated with 1.5 μg/ml puromycin for 7 days. To generate Mthfd2 and G6pdx knockout (KO) N2a cell lines, puromycin selection (1.5 μg/ml) was performed followed by limiting dilution. Single-cell clones were expanded in 96-well plates for 7 days. The corresponding protein expression after gene editing was detected by immunoblotting. The sequences of the sgRNAs used in this study were presented in S2 Table.

### Untargeted metabolomics of mouse brain tissues

For untargeted metabolomics with targeted MRM-based quantification, six-week-old female C57BL/6 mice were intracranially injected with 200 PFU of JEV P3 strain or equal volume DMEM. At 5 days post-infection, the mouse brains were harvested and then subjected to metabolic analysis by using LC-MS. Briefly, each mouse brain was multi-point sampled and accurately weighed to 20 mg and then homogenized in ice-cold 80% LC-MS-grade methanol in water and vortexed for 5 minutes (min). Insoluble material was pelleted by centrifugation at 12000 × g for 10 min. The metabolite extract from the supernatants was subsequently dried down under nitrogen gas for LC-MS analysis.

Chromatographic separation was performed on a Waters ACQUITY UPLC HSS T3 C18 column (1.8 μm, 2.1 mm × 100 mm) using an ExionLC AD UPLC system coupled to a TripleTOF 6600 mass spectrometer (AB SCIEX). The mobile phases consisted of water containing 0.1% formic acid and acetonitrile containing 0.1% formic acid. Mass spectra were acquired in information-dependent acquisition (IDA) mode under both positive and negative electrospray ionization conditions. Qualitative analysis was performed based on retention time (RT), precursor ion information, and MS/MS fragmentation spectra of the detected metabolites. Metabolite identification was achieved using a self-built database together with public databases, including HMDB, KEGG, MoNA, and MassBank. For quantitative analysis, multiple reaction monitoring (MRM) was performed using a QTRAP LC-MS/MS system (AB SCIEX) operating in triple quadrupole mode. QC samples prepared by pooling all sample extracts were injected periodically throughout the analytical run to monitor instrument stability. Chromatographic peak areas were integrated using MultiQuant software (AB SCIEX) and normalized to sample weight. The normalized metabolite abundance data are provided in S3 Table.

### Targeted metabolomics of central carbon metabolism in N2a cells

N2a cells, cultured in 10 cm dishes for intracellular metabolite extraction, were washed twice with ice-cold PBS and frozen in liquid nitrogen to halt metabolic enzyme activity. The intracellular metabolites were then extracted with ice-cold 80% methanol. The extracts were collected by vortexing and shaking for 15 min and centrifuging at 12,000 rpm at 4°C for 10 min. Metabolites were detected by MetWare (Wuhan, China) using the AB Sciex QTRAP 6500 LC-MS/MS platform.

Chromatographic separation was performed on a Waters ACQUITY H-Class UPLC system with an ACQUITY UPLC BEH Amide column (1.7 μm, 2.1 × 100 mm). Mobile phase A was water containing 10 mM ammonium acetate and 0.3% ammonium hydroxide; mobile phase B was 90% acetonitrile. The gradient was: 0–1.2 min, 95% B; 8 min, 70% B; 9–11 min, 50% B; 11.1–15 min, 95% B. Flow rate was 0.4 mL/min, column temperature 40°C, injection volume 2 μL. Detection was performed on an AB Sciex QTRAP 6500 + mass spectrometer with an ESI Turbo Ion-Spray source in both positive and negative modes. Source parameters: temperature 550°C; ion spray voltage 5500 V (positive) / −4500 V (negative); curtain gas 35 psi; GS1/GS2 50 psi. Data were acquired in MRM mode with optimized declustering potential and collision energy for each transition. Analyst 1.6.3 and MultiQuant 3.0.3 software were used for data acquisition and peak integration.

Metabolites were annotated based on retention time and MRM transitions using authentic standards and the MetWare database. Quantification was performed using external standard calibration curves. QC samples, prepared by pooling equal volumes of all sample extracts, were injected every ten runs. Metabolite concentrations were normalized to sample protein content. The detected metabolites spanning central carbon and nucleotide metabolism are listed in S4 Table.

### [U-^13^C] Glucose isotope tracing

N2a cells were seeded in 6 cm dishes at a density of 1.5 × 10^6^ cells/dish. Following 18 h of cell adherence, the cells were infected with JEV at a multiplicity of infection (MOI) of 5 for 1.5 h. After three washes with PBS, the cells were subsequently cultured in glucose-free DMEM medium (Gibco) supplemented with 25 mM [U-^13^C] glucose for 24 h. Then, cells were gently washed three times with PBS and lysed with pre-chilled 80% LC-MS grade methanol at -80 °C for 2 h. The metabolite-containing supernatant, collected after centrifugation at 14,000 × g for 20 min at 4 °C, was dried under a gentle stream of nitrogen at room temperature. The incorporation of ^13^C into CCM was determined using Ultra High-Performance Liquid Chromatography -High-Resolution Mass Spectrometry (UHPLC-HRMS). Chromatographic separation was performed on an Ultimate 3000 UHPLC system (Thermo Fisher, USA) equipped with a Waters BEH C18 column (2.1mm × 100 mm, 1.7 μm). Simultaneously, the mass spectrometry was conducted using a Q Exactive Hybrid Quadrupole-Orbitrap Mass Spectrometer (Thermo Fisher, USA). Metabolite levels in each sample were normalized to their corresponding protein concentrations. The data from the [U-^13^C] Glucose metabolic flux analysis have been deposited in S5 Table.

### Analysis of bulk RNA-sequencing and single-cell RNA-sequencing data

The bulk RNA-sequencing and single-cell RNA-sequencing (scRNA-seq) data used in this study were sourced from our previous research. The datasets generated by Li et al. [27] and Yang et al. [34] are publicly accessible in the Gene Expression Omnibus database under accession numbers GSE94789 and GSE237915, respectively. For our analysis, we retrieved the expression data directly from the original publication and the GEO repository. Subsequently, the expression levels of metabolic enzyme genes were plotted in a heatmap created using the ComplexHeatmap R package (version 2.24.1).

### Inhibitor treatment *in vitro*

N2a cells were infected with JEV at a MOI of 5 in serum-free medium. After 1.5 h for virus absorption, the cell supernatant was replaced with fresh DMEM containing 2% FBS. The inhibitor at an appropriate dosage, determined by a CCK-8 cytotoxicity assay with SuperKine Maximum Sensitivity Cell Counting Kit-8 (Abkkine, BMU106), was added to the maintenance medium of the cell culture. Supernatants and cells were collected at specific time points. The viral titer and RNA abundance were determined using plaque assays and qRT-PCR, respectively.

### Hematoxylin-eosin and immunohistochemistry staining

The harvested mouse brains were fixed overnight in 4% paraformaldehyde (PFA) at 4 °C. A minimum of three biological replicates per experimental group were paraffin-embedded and sectioned to a thickness of 5 μm. The pathological changes in the tissues were evaluated using hematoxylin and eosin (H&E) staining, following standard protocols.

For the detection of JEV E protein expression and microglial activation via immunohistochemistry (IHC) staining, sections were incubated with 3% hydrogen peroxide (H₂O₂) for 30 min to eliminate endogenous peroxidase activity. Subsequently, heat-mediated epitope recovery was performed in citrate buffer (pH 6.0) at 96°C for 30 min. The sections were then blocked with 5% bovine serum albumin (BSA) for 1 h at room temperature, followed by an overnight incubation at 4°C with a mouse anti-IBA-1 mAb (Servicebio, GB15105) or anti-JEV-E mAb (prepared in our lab). After washing with PBS-T, the sections were incubated with horseradish peroxidase-conjugated sheep anti-mouse IgG secondary antibody (Servicebio, GB23301) for 45 min. Finally, signal visualization was achieved using a 3,3’-diaminobenzidine substrate with hematoxylin counterstaining.

All stained sections were digitally scanned with a Leica Apero CS2 slide scanning system for high-resolution analysis of neuropathological features.

### Ag2037 administration in mice

Six-week-old female C57BL/6 mice were randomly assigned to four groups: group 1 consisted of mice injected intravenously with 100 µL DMEM, followed by intravenous injection with 100 µL of 10% DMSO and 90% (20% SBE-β-CD) in saline at 3 and 6 days post-mock infection (Mock+Vehicle, n = 20); group 2 included mock-infected mice i.v. injected with 15 mg/kg of Ag2037 diluted in 100 µL of 10% DMSO and 90% (20% SBE-β-CD) in saline at 3 and 6 days post-mock infection (Mock+Ag2037, n = 20); group 3 received an intravenous injection of 10^5^ PFU of the JEV P3 strain in 100 μL of DMEM, followed by intravenous injection of the vehicle at 3 and 6 days post-infection (dpi) (JEV + Vehicle, n = 20); group 4 consisted of mice injected with 10^5^ PFU of JEV, followed by treatment with 15 mg/kg of Ag2037 (JEV + Ag2037, n = 20). Mice were monitored daily for 21 days post-infection for changes in weight, symptoms and mortality.

### Statistical analysis

All experiments in this study were conducted at least three times under similar conditions. Data analysis was performed using GraphPad Prism, version 9 (GraphPad Software, San Diego, USA). Results are presented as the mean ± standard error of the mean (SEM). Statistical differences between experimental groups were performed using two-tailed Student’s t-test, one-way or two-way ANOVA. For all statistical significance indications in this manuscript, ****, p < 0.0001; ***, p < 0.001; **, p < 0.01; *, p < 0.05, and ns, no significance.

## Supporting information

S1 Fig Metabolic profiling of JEV-infected mouse brains and N2a cells. (A) Principal component analysis (PCA) score plot of metabolomic profiles from mock-infected and JEV-infected mouse brains (n = 5). (B) Volcano plot displaying the significance versus magnitude of change of metabolites. Significantly altered metabolites (VIP > 1, p value < 0.05, |Log2FC| > 1) are highlighted in red (upregulated) and blue (downregulated). (C) Heatmap of Z-scores showing the relative abundance of KEGG-annotated differentially abundant metabolites in mock and JEV-infected brains at 5 dpi. (D) PCA score plot of metabolomic profiles from mock-infected (n = 3) and JEV-infected (n = 3) N2a cells.
(TIF)

S2 Fig [U-¹³C] Glucose tracing analysis of central carbon and nucleotide metabolism in JEV-infected N2a cells. (A) Representative mass isotopologue distributions (M + 0 to M + 6) for key TCA cycle intermediates, which illustrate diverse labeling patterns resulting from isotopic equilibration through multiple turns of the cycle. (B) Normalized peak areas of isotopologues for TCA cycle intermediates from mock- and JEV-infected N2a cells at 24 hpi. (C) Normalized peak areas of isotopologues for glycolytic end products pyruvate (M + 3) and lactate (M + 0 to M + 3) from mock- and JEV-infected N2a cells at 24 hpi. (D) Relative distribution of NTPs isotopologues from mock- and JEV-infected N2a cells at 24 hpi. (E) Normalized peak areas of partially labeled (M + 1 to M + 4) and fully labeled (M + 6 to M + 10) NMP species from mock- and JEV-infected N2a cells at 24 hpi. (F) Normalized peak areas of M + 5 labeled phosphoribosyl pyrophosphate (PRPP), a key precursor for *de novo* nucleotide synthesis, from mock- and JEV-infected N2a cells at 24 hpi. Data are presented as mean ± s.e.m. (n = 3). Statistical significance was determined using two-tailed unpaired Student’s t-tests (*p < 0.05, **p < 0.01, ***p < 0.001, ****p < 0.0001).
(TIF)

S3 Fig Nucleotide composition analysis of JEV P3 strain genome reveals a purine bias. (A) Percentages of individual nucleotides (A, U, C, G) in the JEV P3 genome (GenBank: U47032.1). (B) Combined percentages of purines (A + G) and pyrimidines (C + U). Genomic analysis was performed using EditSeq (DNAstar).
(TIF)

S4 Fig Transcriptional upregulation of DNPB, PPP and 1C metabolism enzymes upon JEV infection in neurons and TM4 cells. (A-D) Relative mRNA levels of core DNPB enzymes in JEV infected neurons and TM4 cells. N2a cells were either infected or mock-infected with JEV at an MOI of 5 (A) or 1 (B). The cells were collected at 24 and 36 hpi for high-dose infection (A) or at 48 hpi for low-dose infection (B). In addition, mouse primary neurons were infected or mock‑infected with JEV at an MOI of 1 and collected at 36 hpi (C). TM4 cells were infected or mock‑infected with JEV at an MOI of 1 and collected at 36 and 48 hpi (D). The mRNA levels of core DNPB enzymes were determined using qRT-PCR. (E) Analysis of single-cell RNA-seq data from brains of JEV-infected mice. Based on the abundance of viral genome, the neuronal populations were classified into 5 groups: mock-infected (Mock), JEV-exposed but viral genome-negative (JEV-N), viral genome-low (JEV-L), viral genome-medium (JEV-M), and viral genome-high (JEV-H). The expression dynamics of DNPB enzymes across these neural populations were presented. (F–H) Relative mRNA levels of core PPP enzymes in JEV infected neurons and TM4 cells. N2a cells were infected or mock‑infected with JEV at an MOI of 5 and collected at 36 hpi (F). Mouse primary neurons were infected or mock‑infected with JEV at an MOI of 1 and collected at 36 hpi (G). TM4 cells were infected or mock‑infected with JEV at an MOI of 1 and collected at 36 and 48 hpi (H). The mRNA levels of the indicated PPP enzymes were quantified by qRT‑PCR. (I) The scRNA-seq profiling of PPP-related genes in neurons from brains of JEV and mock-infected mice. (J-L) Relative mRNA levels of core 1C metabolism enzymes in JEV infected neurons and TM4 cells. N2a cells were infected or mock‑infected with JEV at an MOI of 5 and collected at 36 hpi (J). Mouse primary neurons were infected or mock‑infected with JEV at an MOI of 1 and collected at 36 hpi (K). TM4 cells were infected or mock‑infected with JEV at an MOI of 1 and collected at 36 and 48 hpi (L). The mRNA levels of 1C metabolism enzymes were quantified using qRT-PCR. (M) The scRNA-seq analysis showing compartmentalized expression of cytosolic (e.g., SHMT1, MTHFD1) and mitochondrial (e.g., MTHFD2, SHMT2) 1C enzymes across neurons with differing viral loads. Data are presented as mean ± s.e.m of 3 independent biological replicates (n = 3). Statistical significance was determined by two-tailed unpaired Student’s t-test (*p < 0.05, **p < 0.01, ***p < 0.001).
(TIF)

S5 Fig Cytotoxicity profiling of metabolic inhibitors via CCK-8 assay. Cytotoxicity of pharmacological agents on N2a cells. N2a cells were treated with indicated concentrations of MPA (A), Ag2037 (B), RRx-001(C), 6-AN (D), DS18561882 (E), or corresponding vehicle. The cell viability was determined at 24 h post-treatment and normalized to vehicle-treated controls. Data represent mean ± s.e.m. from 3 independent biological replicates. The dashed line indicates the 90% viability threshold used to define acceptable cytotoxicity in subsequent *in vitro* experiments.
(TIF)

S1 Table Oligonucleotide primers for qPCR analysis. (DOCX)

S2 Table Designed sgRNA sequences for CRISPR/Cas9 gene editing. (DOCX)

S3 Table Untargeted metabolomics data. (XLSX)

S4 Table Targeted metabolomics data. (XLSX)

S5 Table [U-^13^C] Glucose metabolic flux data. (XLSX)
