# Supplementary figures and images for: Japanese encephalitis virus hijacks the host purine biosynthetic network to promote viral replication in neurons
Source: PLoS Pathog. 2026 Jul 7;22(7):e1014335. doi: 10.1371/journal.ppat.1014335 (PMC13340812; doi:10.1371/journal.ppat.1014335)

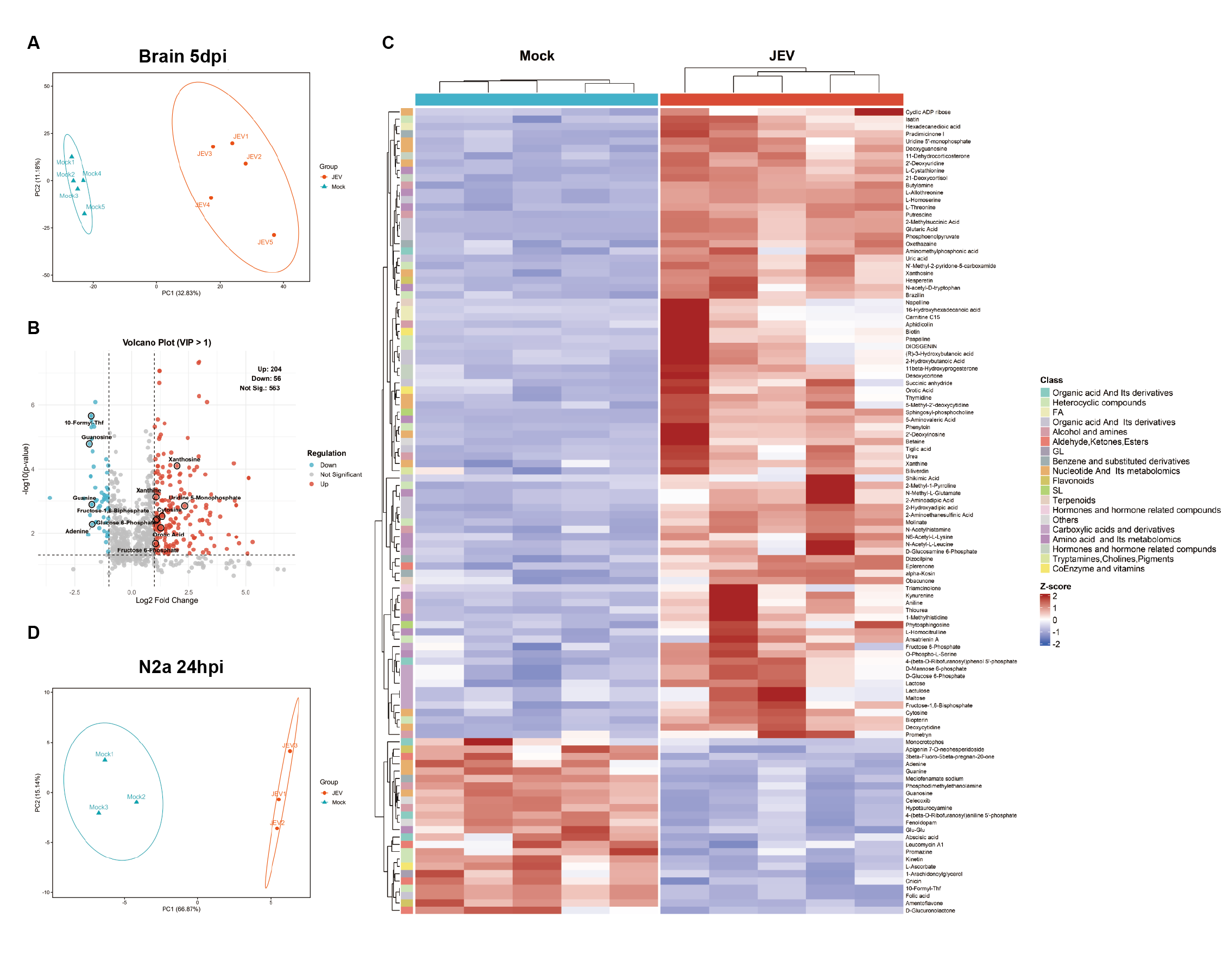

Supplement: S1 Fig — (A) Principal component analysis (PCA) score plot of metabolomic profiles from mock-infected and JEV-infected mouse brains (n = 5). (B) Volcano plot displaying the significance versus magnitude of change of metabolites. Significantly altered metabolites (VIP > 1, p value < 0.05, |Log2FC| > 1) are highlighted in red (upregulated) and blue (downregulated). (C) Heatmap of Z-scores showing the relative abundance of KEGG-annotated differentially abundant metabolites in mock and JEV-infected brains at 5 dpi. (D) PCA score plot of metabolomic profiles from mock-infected (n = 3) and JEV-infected (n = 3) N2a cells. (TIF) [file ppat.1014335.s001.tif]

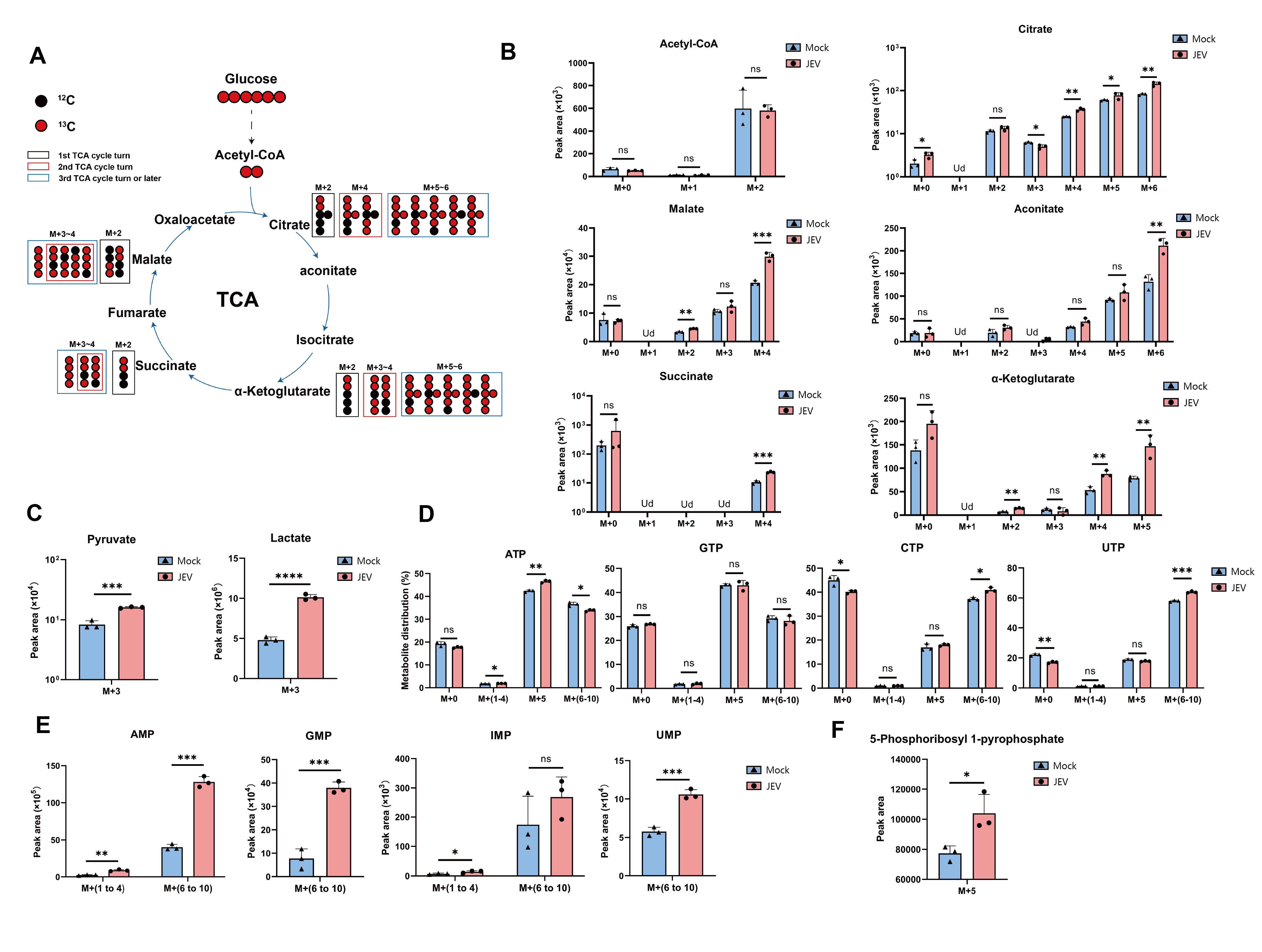

Supplement: S2 Fig — (A) Representative mass isotopologue distributions (M + 0 to M + 6) for key TCA cycle intermediates, which illustrate diverse labeling patterns resulting from isotopic equilibration through multiple turns of the cycle. (B) Normalized peak areas of isotopologues for TCA cycle intermediates from mock- and JEV-infected N2a cells at 24 hpi. (C) Normalized peak areas of isotopologues for glycolytic end products pyruvate (M + 3) and lactate (M + 0 to M + 3) from mock- and JEV-infected N2a cells at 24 hpi. (D) Relative distribution of NTPs isotopologues from mock- and JEV-infected N2a cells at 24 hpi. (E) Normalized peak areas of partially labeled (M + 1 to M + 4) and fully labeled (M + 6 to M + 10) NMP species from mock- and JEV-infected N2a cells at 24 hpi. (F) Normalized peak areas of M + 5 labeled phosphoribosyl pyrophosphate (PRPP), a key precursor for de novo nucleotide synthesis, from mock- and JEV-infected N2a cells at 24 hpi. Data are presented as mean ± s.e.m. (n = 3). Statistical significance was determined using two-tailed unpaired Student’s t-tests (*p < 0.05, **p < 0.01, ***p < 0.001, ****p < 0.0001). (TIF) [file ppat.1014335.s002.tif]

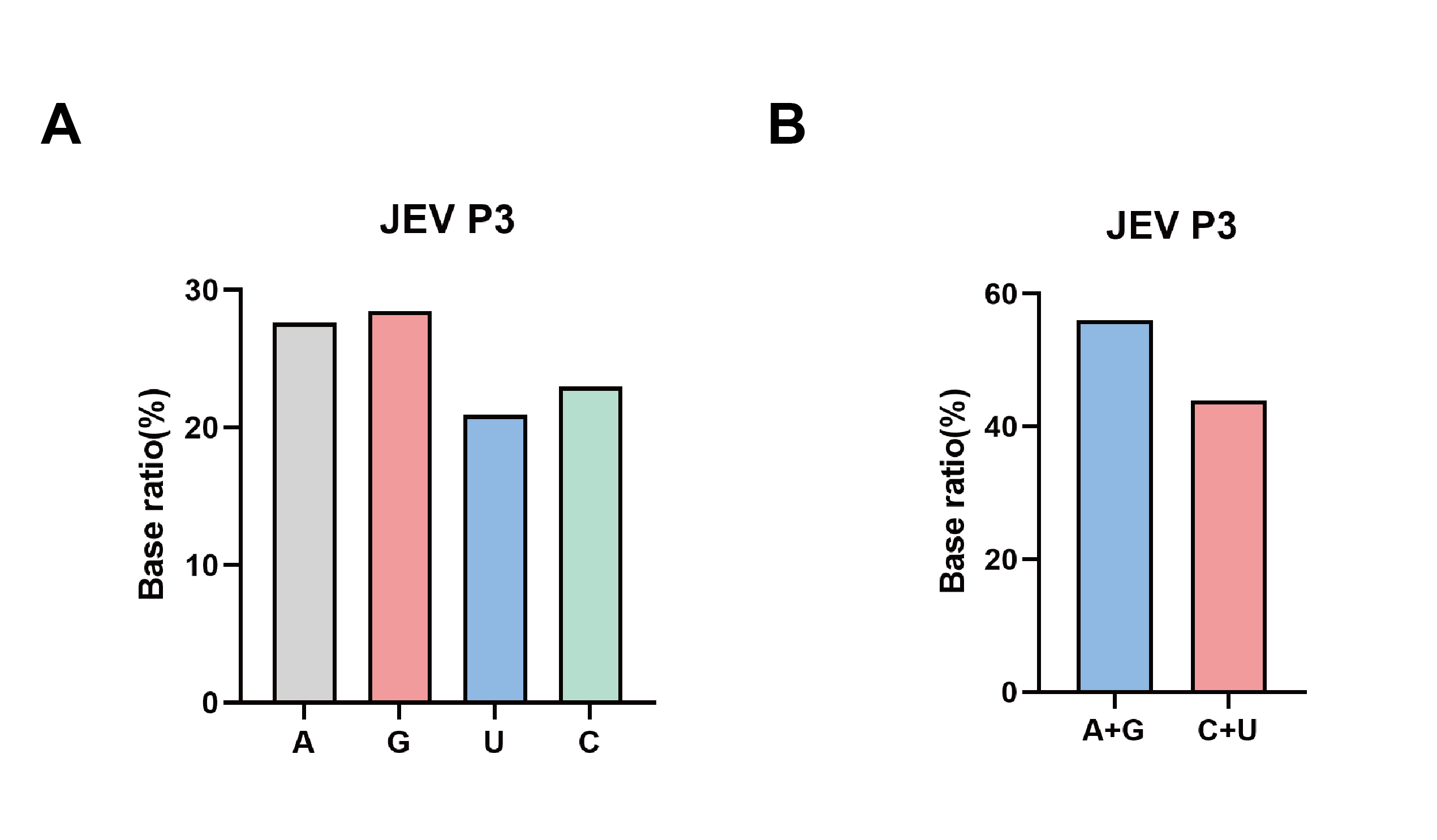

Supplement: S3 Fig — (A) Percentages of individual nucleotides (A, U, C, G) in the JEV P3 genome (GenBank: U47032.1). (B) Combined percentages of purines (A + G) and pyrimidines (C + U). Genomic analysis was performed using EditSeq (DNAstar). (TIF) [file ppat.1014335.s003.tif]

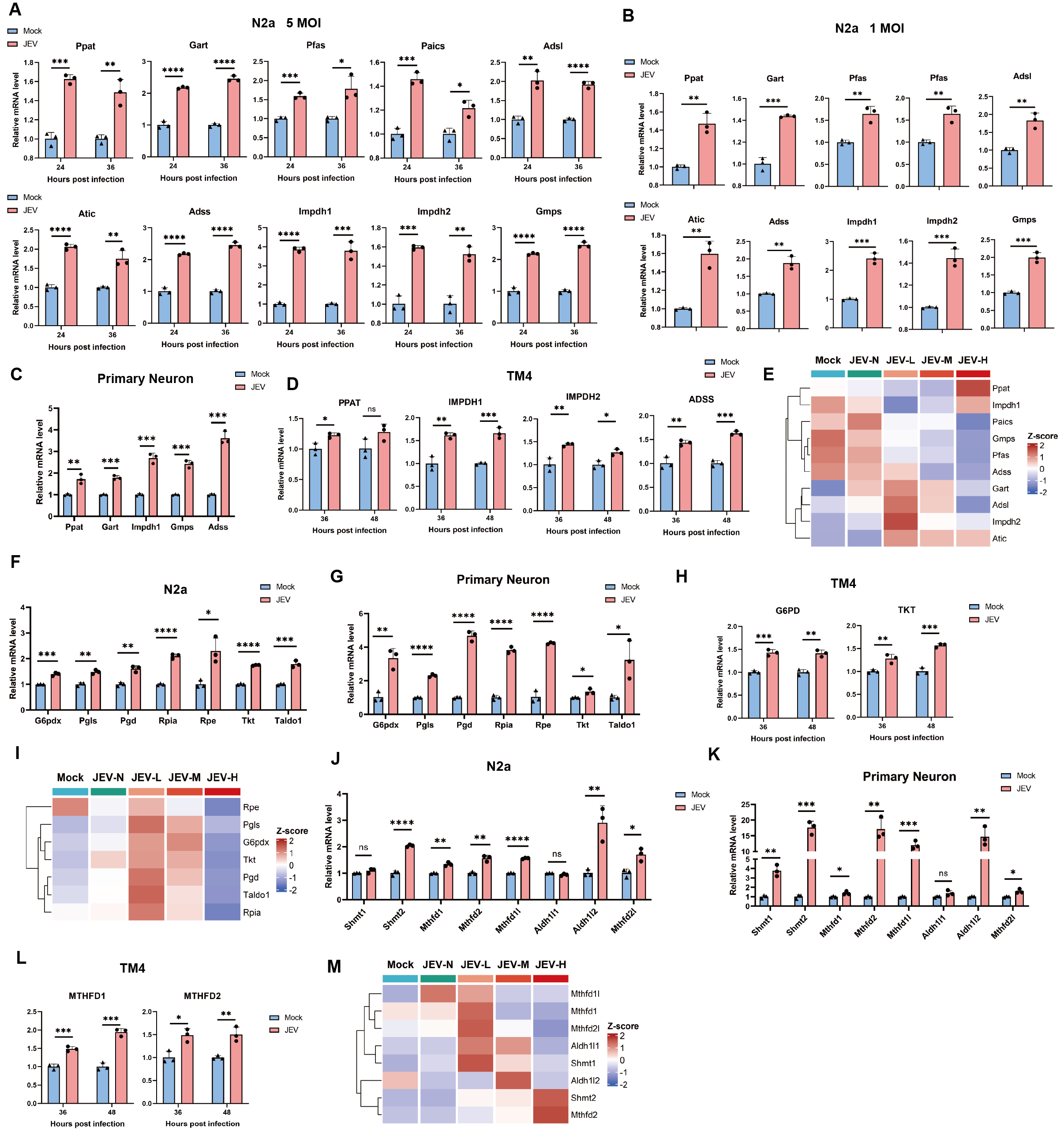

Supplement: S4 Fig — (A-D) Relative mRNA levels of core DNPB enzymes in JEV infected neurons and TM4 cells. N2a cells were either infected or mock-infected with JEV at an MOI of 5 (A) or 1 (B). The cells were collected at 24 and 36 hpi for high-dose infection (A) or at 48 hpi for low-dose infection (B). In addition, mouse primary neurons were infected or mock‑infected with JEV at an MOI of 1 and collected at 36 hpi (C). TM4 cells were infected or mock‑infected with JEV at an MOI of 1 and collected at 36 and 48 hpi (D). The mRNA levels of core DNPB enzymes were determined using qRT-PCR. (E) Analysis of single-cell RNA-seq data from brains of JEV-infected mice. Based on the abundance of viral genome, the neuronal populations were classified into 5 groups: mock-infected (Mock), JEV-exposed but viral genome-negative (JEV-N), viral genome-low (JEV-L), viral genome-medium (JEV-M), and viral genome-high (JEV-H). The expression dynamics of DNPB enzymes across these neural populations were presented. (F–H) Relative mRNA levels of core PPP enzymes in JEV infected neurons and TM4 cells. N2a cells were infected or mock‑infected with JEV at an MOI of 5 and collected at 36 hpi (F). Mouse primary neurons were infected or mock‑infected with JEV at an MOI of 1 and collected at 36 hpi (G). TM4 cells were infected or mock‑infected with JEV at an MOI of 1 and collected at 36 and 48 hpi (H). The mRNA levels of the indicated PPP enzymes were quantified by qRT‑PCR. (I) The scRNA-seq profiling of PPP-related genes in neurons from brains of JEV and mock-infected mice. (J-L) Relative mRNA levels of core 1C metabolism enzymes in JEV infected neurons and TM4 cells. N2a cells were infected or mock‑infected with JEV at an MOI of 5 and collected at 36 hpi (J). Mouse primary neurons were infected or mock‑infected with JEV at an MOI of 1 and collected at 36 hpi (K). TM4 cells were infected or mock‑infected with JEV at an MOI of 1 and collected at 36 and 48 hpi (L). The mRNA levels of 1C metabolism enzymes were quantifi [file ppat.1014335.s004.tif]

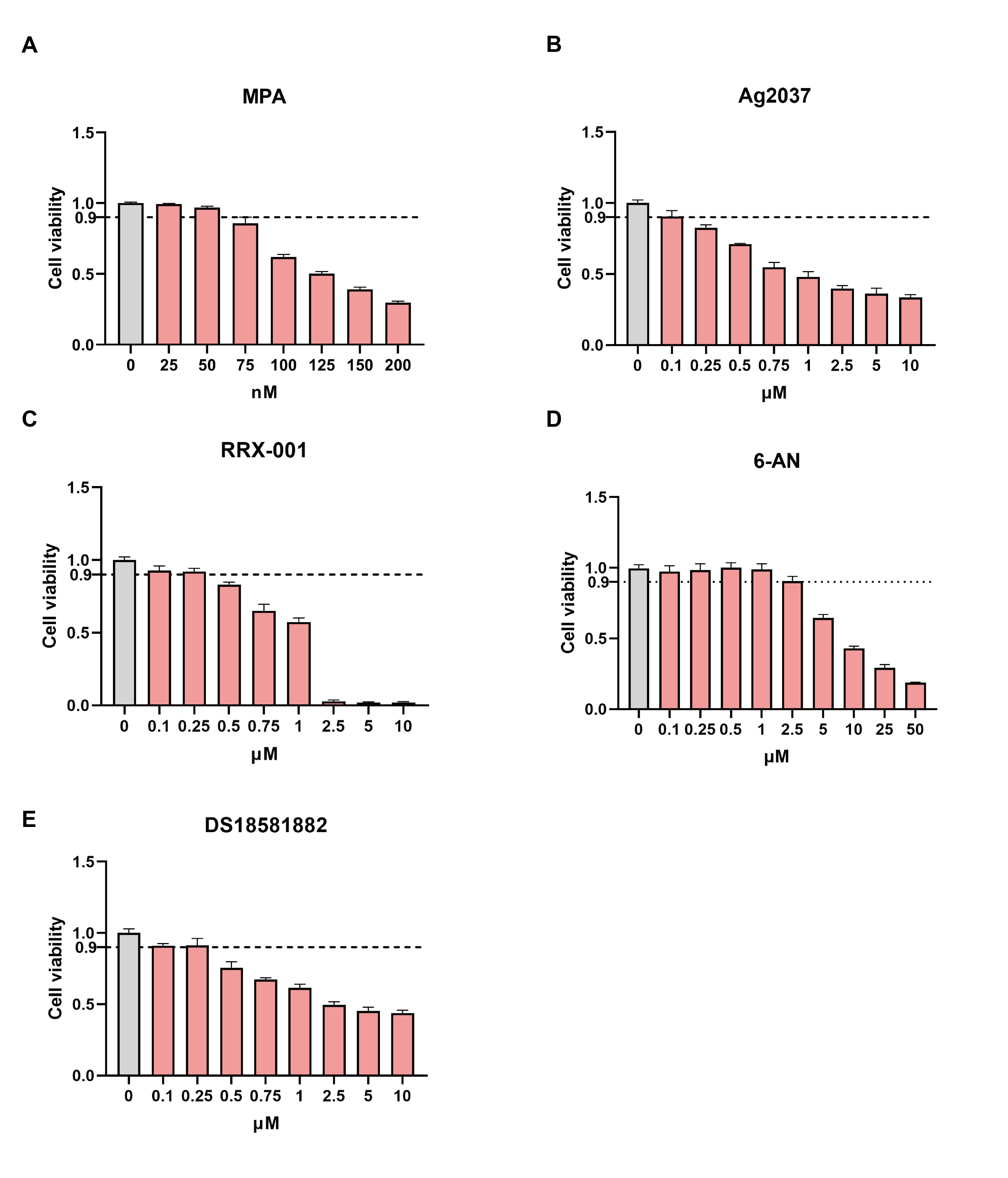

Supplement: S5 Fig — Cytotoxicity of pharmacological agents on N2a cells. N2a cells were treated with indicated concentrations of MPA (A), Ag2037 (B), RRx-001(C), 6-AN (D), DS18561882 (E), or corresponding vehicle. The cell viability was determined at 24 h post-treatment and normalized to vehicle-treated controls. Data represent mean ± s.e.m. from 3 independent biological replicates. The dashed line indicates the 90% viability threshold used to define acceptable cytotoxicity in subsequent in vitro experiments. (TIF) [file ppat.1014335.s005.tif]
