# Supplementary material for: Japanese encephalitis virus hijacks the host purine biosynthetic network to promote viral replication in neurons
Source: PLoS Pathog. 2026 Jul 7;22(7):e1014335. doi: 10.1371/journal.ppat.1014335 (PMC13340812; doi:10.1371/journal.ppat.1014335)
Supplement: S1 Table — (DOCX) [file ppat.1014335.s006.docx]

**S1 Table. Oligonucleotide primers for qPCR analysis.**

| **Target** | **Name** | **Sequence (5’-3’)** |
| --- | --- | --- |
| DNPB | Ppat-F | GAGCTGGAGGAGTTGGGGAT |
| DNPB | Ppat-R | CTCTCCTGACCTCGGTGCTG |
| DNPB | Gart-F | ACCGCCATGAAATCCCAACT |
| DNPB | Gart-R | GCAGGAAAGTTCGCGCTTG |
| DNPB | Pfas-F | AGAACTGAGTGTGCGACAGG |
| DNPB | Pfas-R | CACAGAGCGATCCACCTTGT |
| DNPB | Paics-F | CGGGCAGAAGCAATGGTCTA |
| DNPB | Paics-R | ACAGCCAATACCACTGGGC |
| DNPB | Atic-F | ATGACCTCTACCCGACCCTC |
| DNPB | Atic-R | ACCATCCGACACTTCTCTGG |
| DNPB | Adss-F | TACCTCATTTCCCAGCAAACCA |
| DNPB | Adss-R | TTTTGTGCGTTGACAGGTAGC |
| DNPB | Adsl-F | GGACAGACGTACACACGGAA |
| DNPB | Adsl-R | ATCGCACTGGAGCCAATCTG |
| DNPB | Impdh1-F | AGCTGATGAAGTGGACCTGAC |
| DNPB | Impdh1-R | TCATTGGCCTGGAACTCTGG |
| DNPB | Impdh2-F | ACCCCATTGGTTTCCTCACC |
| DNPB | Impdh2-R | CAATACCTCCTGTAAGCGCCA |
| DNPB | Gmps-F | GCCCTGTGCAACGGAGATT |
| DNPB | Gmps-R | GCTCCTTCGTAATGGTGAGAAC |
| PPP | G6pdx-F | CACAGTGGACGACATCCGAAA |
| PPP | G6pdx-R | AGCTACATAGGAATTACGGGCAA |
| PPP | Pgls-F | CCAGGTCCTTACCATCAATCCT |
| PPP | Pgls-R | AGGGAAGAGCGAACAGGTATG |
| PPP | Pgd-F | ATGGCCCAAGCTGACATTG |
| PPP | Pgd-R | GCACAGACCACAAATCCATGAT |
| PPP | Rpia-F | AAGGCCGAGGAGGCTAAGAA |
| PPP | Rpia-R | CTTTCAGCTATTCGCTGCACA |
| PPP | Rpe-F | GCACCTGGATGTAATGGACGG |
| PPP | Rpe-R | CCTGGCCTAGCTGCTTTCG |
| PPP | Tkt-F | ATGGAAGGTTACCATAAGCCAGA |
| PPP | Tkt-R | TGCAGCATGATGTGGGGTG |
| PPP | Taldo1-F | GAGCGGATGCTCACGGAAC |
| PPP | Taldo1-R | TGCAATTAGGACCCAGCTCAA |
| 1CM | Shmt1-F | TCCACGCTCCTAATACAAGGC |
| 1CM | Shmt1-R | TGATGCTGTAAACCTCGGCA |
| 1CM | Shmt2-F | GACCACTCGGCCTCTGC |
| 1CM | Shmt2-R | TCCCACATCTCAGGGTCACT |
| 1CM | Mthfd1-F | TAACTCGGATGCAGGAGCAG |
| 1CM | Mthfd1-R | AGTGGCTTTGATCCCAATCTCT |
| 1CM | Mthfd2-F | TCCTTGTTGTCTGCGTTGGC |
| 1CM | Mthfd2-R | TGACAACGGCTTCATTTCGCA |
| 1CM | Mthfd1l-F | GCGGAGAGGATGAGATCATAGA |
| 1CM | Mthfd1l-R | GTCACCCCGTCCACATCTT |
| 1CM | Mthfd2l-F | CGGCCGGAGACACGAAG |
| 1CM | Mthfd2l-R | CGAGAGCGATCCATGATTCCA |
| 1CM | Aldh1l1-F | CGTCTTTGACCTTGGGTGCCT |
| 1CM | Aldh1l1-R | TGTCTGGGATGGTGAACACAC |
| 1CM | Aldh1l2-F | TCTGCCATCAACTGGACACT |
| 1CM | Aldh1l2-R | ACCATCATCTGCCCAGAAAACA |
| JEV | JEV-E-F | TGGTTTCATGACCTCGCTCTC |
| JEV | JEV-E-R | CCATGAGGAGTTCTCTGTTTCT |
| JEV | JEV-Probe | CCTGGACGCCCCCTTCGAGCACAGCGT |
| Cytokine | TNF-α-F | TGTCTCAGCCTCTTCTCATTCC |
| Cytokine | TNF-α-R | TTAGCCCACTTCTTTCCCTCAC |
| Cytokine | CCL-5-F | TGCCCACGTCAAGGAGTATTTC |
| Cytokine | CCL-5-R | AACCCACTTCTTCTCTGGGTTG |
| Cytokine | IL-1β-F | AACCTGCTGGTGTGTGACGTTC |
| Cytokine | IL-1β-R | CAGCACGAGGCTTTTTTGTTGT |
| Cytokine | CCL-2-F | CGGCGAGATCAGAACCTACAAC |
| Cytokine | CCL-2-R | GGCACTGTCACACTGGTCACTC |
| Cytokine | IFN-β-F | CTCCACCACAGCCCTCTC |
| Cytokine | IFN-β-R | CATCTTCTCCGTCATCTCCATAG |
| Cytokine | IL-6-F | AATGAGGAGACTTGCCTGGT |
| Cytokine | IL-6-R | GCAGGAACTGGATCAGGACT |
| Reference gene | β-actin-F | CACTGCCGCATCCTCTTCCTCCC |
| Reference gene | β-actin-R | CAATAGTGATGACCTGGCCGT |
