# Supplementary material for: Japanese encephalitis virus hijacks the host purine biosynthetic network to promote viral replication in neurons
Source: PLoS Pathog. 2026 Jul 7;22(7):e1014335. doi: 10.1371/journal.ppat.1014335 (PMC13340812; doi:10.1371/journal.ppat.1014335)
Supplement: S2 Table — (DOCX) [file ppat.1014335.s007.docx]

**S2 Table. Designed sgRNA sequences for CRISPR/Cas9 gene editing.**

| **Target** | **Name** | **Sequence (5’-3’)** |
| --- | --- | --- |
| G6pdx | G6pdxsg | GTCCACTGTGAGTCGTGAGC |
| Tkt | Tktsg1 | TCGTCCTACCACGCCATGGA |
| Tkt | Tktsg2 | GCTTGCGGGATGGCATACAC |
| Mthfd2 | Mthfd2sg | GCTTTCATGTCATTAACGTG |
